# Effects of a community gardening intervention on diet, physical activity, and anthropometry outcomes in the USA (CAPS): an observer-blind, randomised controlled trial

**DOI:** 10.1016/S2542-5196(22)00303-5

**Published:** 2023-01

**Authors:** Jill S Litt, Katherine Alaimo, Kylie K Harrall, Richard F Hamman, James R Hébert, Thomas G Hurley, Jenn A Leiferman, Kaigang Li, Angel Villalobos, Eva Coringrato, Jimikaye Beck Courtney, Maya Payton, Deborah H Glueck

**Affiliations:** Department of Environmental Studies, University of Colorado Boulder, Boulder, CO, USA (Prof J S Litt PhD, A Villalobos BA, E Coringrato BA); Department of Food Science and Human Nutrition, Michigan State University, East Lansing, MI, USA (K Alaimo PhD); Department of Epidemiology (K K Harrall MS, Prof R F Hamman MD) and Department of Community and Behavioural Health (Prof J A Leiferman PhD), Colorado School of Public Health and Department of Pediatrics (Prof D H Glueck PhD), Lifecourse Epidemiology of Adiposity and Diabetes Center (K K Harrall) University of Colorado School of Medicine (Prof D H Glueck), University of Colorado Anschutz Medical Campus, Aurora, CO, USA; Department of Epidemiology and Biostatistics, Arnold School of Public Health, University of South Carolina, Columbia, SC, USA (Prof J R Hébert MSPH ScD, T G Hurley MS); Department of Health and Exercise Science, College of Health and Human Sciences, Colorado State University, Fort Collins, CO, USA (K Li PhD); Department of Exercise and Sport Science, University of North Carolina at Chapel Hill, Chapel Hill, NC, USA (J B Courtney PhD); Urban Institute, Washington, DC, USA (M Payton BA)

## Abstract

**Background:**

Unhealthy diet, physical inactivity, and social disconnection are important modifiable risk factors for non-communicable and other chronic diseases, which might be alleviated through nature-based community interventions. We tested whether a community gardening intervention could reduce these common health risks in an adult population that is diverse in terms of age, ethnicity, and socioeconomic status.

**Methods:**

In this observer-blind, randomised, controlled trial, we recruited individuals who were on Denver Urban Garden waiting lists for community gardens in Denver and Aurora (CO, USA), aged 18 years or older, and had not gardened in the past 2 years. Participants were randomly assigned (1:1), using a randomised block design in block sizes of two, four, or six, to receive a community garden plot (intervention group) or remain on a waiting list and not garden (control group). Researchers were masked to group allocation. Primary outcomes were diet, physical activity, and anthropometry; secondary outcomes were perceived stress and anxiety. During spring (April to early June, before randomisation; timepoint 1 [T1]), autumn (late August to October; timepoint 2 [T2]), and winter (January to March, after the intervention; timepoint 3 [T3]), participants completed three diet recalls, 7-day accelerometry, surveys, and anthropometry. Analyses were done using the intention-to-treat principle (ie, including all participants randomly assigned to groups, and assessed as randomised). We used mixed models to test time-by-intervention hypotheses at an α level of 0·04, with T2 and T3 intervention effects at an α level of 0·005 (99·5% CI). Due to potential effects of the COVID-19 pandemic on outcomes, we excluded all participant data collected after Feb 1, 2020. This study is registered with ClinicalTrials.gov, NCT03089177, and data collection is now complete.

**Findings:**

Between Jan 1, 2017, and June 15, 2019, 493 adults were screened and 291 completed baseline measures and were randomly assigned to the intervention (n=145) or control (n=146) groups. Mean age was 41·5 years (SD 13·5), 238 (82%) of 291 participants were female, 52 (18%) were male, 99 (34%) identified as Hispanic, and 191 (66%) identified as non-Hispanic. 237 (81%) completed measurements before the beginning of the COVID-19 pandemic. One (<1%) participant in the intervention group had an adverse allergic event in the garden. Significant time-by-intervention effects were observed for fibre intake (p=0·034), with mean between-group difference (intervention minus control) at T2 of 1·41 g per day (99·5% CI –2·09 to 4·92), and for moderate-to-vigorous physical activity (p=0·012), with mean between-group difference of 5·80 min per day (99·5% CI –4·44 to 16·05). We found no significant time-by-intervention interactions for combined fruit and vegetable intake, Healthy Eating Index (measured using Healthy Eating Index-2010), sedentary time, BMI, and waist circumference (all p>0·04). Difference score models showed greater reductions between T1 and T2 in perceived stress and anxiety among participants in the intervention group than among those in the control group.

**Interpretation:**

Community gardening can provide a nature-based solution, accessible to a diverse population including new gardeners, to improve wellbeing and important behavioural risk factors for non-communicable and chronic diseases.

**Funding:**

American Cancer Society, University of Colorado Cancer Centre, University of Colorado Boulder, National Institutes of Health, US Department of Agriculture National Institute of Food and Agriculture, Michigan AgBioResearch Hatch projects.

## Introduction

Cancer, cardiovascular disease, and diabetes remain some of the most important public health challenges worldwide.^1^ The American Cancer Society, WHO, the International Agency for Research on Cancer, the World Heart Federation, and other organisations report that, in addition to smoking, major modifiable risk factors for chronic diseases include poor diet (including low fruit, vegetable, and fibre intake) and physical inactivity.^2^

Community gardens (also known as allotment gardens) are a promising nature-based lifestyle intervention that might promote active, healthy, and socially engaged living, reducing risk factors for chronic diseases. Community gardens can be a nature-based solution that influences emotional, social, and environmental factors that interplay with individual behaviours. Community gardens are places where people garden collectively. The act of community gardening also offers structural opportunities for eating healthy diets and being active and a “setting for the mind” by providing a refuge from everyday stressors^3^ and a way to enhance ecological connections. The components of a holistic community gardening intervention include proximity to nature, access to tools to grow, consume, and share food, opportunities for outdoor physical activity, a network of neighbours with a shared interest in gardening, and an activity that promotes cognitive stimulation and fosters meaningful experiences.^4^ Thus, community gardening networks serve as multicomponent interventions that could reduce risk factors for cancer and other chronic diseases and promote wellbeing worldwide.^5^

Although qualitative and observational evidence show positive effects of community gardening on psychosocial outcomes and fruit and vegetable intake,^5,6^ most of these studies have neither addressed selection bias nor adjusted for confounders.^6^ To our knowledge, three randomised controlled trials of gardening among adults have been published to date, and have considered home gardens but not community gardens. One home-based garden trial showed improvements in fruit and vegetable intake among adult cancer survivors.^7^ Another home-based garden trial improved diet and food security among women in Tanzania.^8^ A home-based gardening trial is underway among Native American families in the Wind River Indian Reservation in Wyoming, USA, with BMI as the primary outcome.^9^

The Community Activation for Prevention Study (CAPS) was designed to study the effect of community gardens on health behaviours and psychosocial outcomes among adults who are diverse in terms of age, ethnicity, and socioeconomic status. Primary outcomes included diet, physical activity, and anthropometry, with secondary outcomes of perceived stress and anxiety.

## Methods

### Study design and participants

The CAPS randomised controlled trial was run at 37 community gardens in Denver and Aurora in Colorado, USA, administered by Denver Urban Gardens (DUG). Garden waiting lists were used as the basis for recruitment of study participants. These waiting lists were pre-existing at each of the gardens included in our study. Before recruitment began, study staff canvassed the surrounding neighbourhoods and partnered with neighbourhood-based organisations to raise awareness about the community gardens and encourage residents to become involved by joining their local community garden waiting list. After 6 weeks of garden promotion activities, study staff, in coordination with DUG staff, invited individuals on the garden waiting lists to join the study. Participants were eligible if they were aged 18 years or older, able to give consent in English or Spanish, had not gardened in the past 2 years, and were willing to not garden during the study period. Only one person per household could participate. Once individuals agreed to participate, study staff obtained written informed consent and conducted the baseline assessments, and participants were randomly assigned within garden waiting lists.

Participant recruitment was done via both face-to-face and media-based approaches (ie, social media, radio, newspapers, newsletters and bulletins). Recruitment was conducted over three waves of data collection, with the aim of recruiting 104 participants per year for 3 years (ie, one wave per year). We aimed to recruit participants from low-income areas. Additional details about the trial design and rationale are described elsewhere.^10,11^

The study protocol was approved by the University of Colorado Boulder Institutional Review Board (UCB IRB; protocol number 16–0644) and monitored by the University of Colorado Cancer Center Data Safety and Monitoring Committee. All study participants provided written informed consent before enrolment and randomisation. All seven primary outcomes were approved on Nov 1, 2016, by the UCB IRB Office of Human Subjects.

This study is registered with ClinicalTrials.gov, NCT03089177. When the trial was originally registered at ClinicalTrials.gov, bodyweight was listed as a primary outcome measure instead of BMI. Additionally, perceived anxiety, a secondary outcome, was approved by the UCB IRB but was not included in the original registration. Finally, in the published protocol of the CAPS,^10^ we provided a description of the methods for assessing diet and physical activity but did not specify explicitly the primary outcomes of combined fruit and vegetable intake, Healthy Eating Index, fibre, moderate-to-vigorous physical activity, and sedentary time. Prespecified diet and physical activity outcomes reported here are listed in the ClinicalTrials.gov registration.

### Randomisation and masking

We used a pseudo-random-number generator (sample function, R studio) with a random seed to choose participant assignments. Participants were randomly assigned (1:1) independently within each community garden waiting list to either the community gardening group (intervention group) or to stay on the waiting list with no gardening (control group). The approach involved permuted block randomisation with varying block sizes. Given the size of each garden waiting list, the algorithm randomly selected blocks of size two, four, or six, so that the total number of participants in all blocks was equal to the number of people on the waiting list for the garden. Assignments were generated by study statisticians (DHG and KKH) who had no contact with participants. Randomised assignments were transmitted to the study coordinator in sealed envelopes. The study coordinator informed participants of their group allocation after baseline data collection. Participants randomly assigned to the control group were not eligible to be re-randomised in a subsequent year. Study participants were not masked to assignment, but study staff conducting assessments, investigators, and statisticians were masked to participant assignments until after data collection was completed and the planned analyses for primary outcomes were finished.

### Procedures

In May of each wave, after the typical last frost in Denver and Aurora, participants randomly assigned to the intervention gardening group were provided a standard community garden plot (average size of 10 m^2^), seeds and seedlings, and an introductory gardening course taught through DUG. Plot fees were covered by the trial. We worked with community garden leaders to secure two to six plots per garden for study participants. A garden might have been included in the study for one wave, two waves, or all three waves. If a garden was not included in subsequent waves, new gardens were recruited to the study. The community garden organisation staff, leaders, and members offered opportunities for social interaction, community building, and mentorship through events, workdays, and classes. After each wave of the trial, when a participant’s trial participation was completed (ie, 1 year of participation), as recompense for agreeing to wait for a year to garden, control participants were offered a garden plot the next growing season, with plot fees, seeds and seedlings, and an introductory gardening course paid for by the trial.

Health surveys, including perceived measures of stress and anxiety, accelerometry, and dietary interviews, were administered to all participants at baseline before the gardening season and before random allocation (April to early June, timepoint 1 [T1]), during autumn harvest (ie, end of August to October; timepoint 2 [T2]), and during the winter (ie, January to March; after the intervention (timepoint 3 [T3]). A retention incentive (US$25 at T1, $50 at T2, and $75 at T3) was offered to all participants who attended each study assessment.

Health visits were done at T1, T2, and T3 at the central offices of DUG in northeast Denver. Participants completed the health visit with staff from whom allocated assignment was concealed. The participants completed 1·5 h of assessments, including the health survey, anthropometric measurements (height, bodyweight, and waist circumference), and the placement of the thigh-mounted accelerometer. Dietary assessments, via telephone, were completed on three randomly selected days by registered dieticians after the in-person health visit. Details of the health visits are reported elsewhere.^10^

We collected demographic variables, including age, sex, self-identified ethnicity, primary language, house hold income, educational attainment, years of previous gardening experience, and smoking status, via survey at T1. Social desirability bias was measured using the short form of the Marlowe-Crowne Social Desirability Scale.^12^

### Outcomes

We had seven prespecified primary outcomes: fibre intake, combined fruit and vegetable intake, Healthy Eating Index, moderate-to-vigorous physical activity, sedentary time, BMI, and waist circumference.

For dietary intake, participants completed three unannounced, randomly timed, telephone-administered 24-h recall interviews with bilingual (English and Spanish) registered dietitians at each of the three data collection timepoints. We used Nutrient Data System for Research software (version 2020; Nutrition Coordinating Center (NCC), University of Minnesota, Minneapolis, MN, USA) to administer the dietary inter views and calculate dietary intake outcomes, including fibre. Estimates of combined fruit and vegetable intake excluded juice, avocados, French fries, and fried onion rings. The Healthy Eating Index (HEI-2010) was calculated on the basis of 13 dietary components, with higher scores indicating better compliance with national dietary guidelines.^13^

For anthropometrics, participant height was measured with a portable stadiometer (Seca 213 Portable Stadiometer; Seca, Hangzhou, China), accurate to the nearest 0·1 cm. Bodyweight was measured with a digital platform scale (Seca 876 Digital Scale; Seca) to the nearest 0·23 kg (0·5 lb). Waist circumference was measured at the superior border of the iliac crest, using a cloth tape to the nearest 0·1 cm, while the participant was standing. BMI was calculated as kg/m^2^.

For physical activity, participants wore thigh-mounted ActivPAL3 accelerometers (PAL Technologies, Glasgow, UK) for 7 days after each assessment timepoint.^14^ Participants with at least 3 valid weekdays and 1 valid weekend day of data were included in the analyses, with a valid day defined as wearing the device for at least 10 h while awake. Average weekend and weekday moderate-to-vigorous physical activity (min per day) and sitting time (min per day) were calculated using valid wear days. Moderate-to-vigorous physical activity was defined as a stepping cadence of at least 75 steps per min,^15^ and sitting time was defined as sitting down for at least 30 min consecutively.

Our secondary outcomes were perceived stress and anxiety. Perceived stress was measured using the Perceived Stress Scale 10 (PSS-10). The range of PSS-10 is between 0 and 40, with lower scores denoting lower stress levels and higher scores higher stress levels.^16^ Perceived anxiety was measured using the General Anxiety Disorder 7 (GAD-7) scale. The range of the GAD-7 is between 0 and 21, with lower scores denoting lower anxiety levels and higher scores denoting higher anxiety levels.^17^ For PSS-10 and GAD-7, a positive difference score between timepoints (ie, score at T2 minus score at T1 or score at T3 minus score at T1) indicated an increase in stress or anxiety from baseline, whereas a negative difference score indicated a decrease in stress or anxiety levels from baseline.

### Statistical analysis

We calculated the sample size using a separate power analysis for each of the three primary outcome categories: diet, physical activity, and anthropometry. The final sample size was chosen as the maximum of the three sample sizes found to provide power of at least 80% for each outcome category. We did not do a formal power analysis for perceived stress and anxiety because they were secondary outcomes.^10^ We calculated the power of the study assuming a 30% loss to follow-up, such that if we recruited 312 participants for 30 gardens, we would be left with 218 participants across 30 gardens.

We did all analyses under the intention-to-treat principle (ie, included all participants randomly assigned to groups), and analysed them as randomised. Because the COVID-19 pandemic affected data collection and might have had an effect on health behaviours, the study team agreed to exclude wave 3, T3 data from the analysis—ie, data collected after Feb 1, 2020. The decision was made a priori, while assessors and investigators were still masked to group assignments. We tested time-by-intervention hypotheses at an α level of 0·04 and intervention effects at T2 and T3 at an α level of 0·005 (99·5% CI), for a total type 1 error rate of (0·04 + 0·005 + 0·005 =) 0·05 per primary outcome. A significant time-by-intervention interaction indicated that the pattern of means over time for the intervention group differed from that of the control group.

Participants with and without missing data were compared overall and stratified by intervention assignment using Rao-Scott χ^2^ tests (appendix p 2). We assessed the balance of demographic features across the two groups using the Rao-Scott test for categorical demographic variables and mixed models accounting for clustering within garden waiting lists for continuous demographic variables.

For dietary outcomes, we used general linear mixed models to assess time-by-intervention interaction. The outcome at each timepoint (T1, T2, and T3) was the mean of the response for the 24-h recalls. We modelled correlations using the Kronecker-product covariance matrix, with an unstructured model for the longitudinal repeated measures, and the best-fitting choice of one of two parameterisations to account for clustering: (1) a model with a waiting list random effect only, and (2) a model with a random term for assignment to gardening, nested within the waiting list. For all mixed models, we used jackknife studentised residuals to test modelling assumptions. We assessed significance using the Wald test and Kenward-Roger degrees of freedom. In preliminary modelling, we assessed wave effects and found no difference in outcome by wave. This indicated that data could be combined across the 3 years of recruitment, as we present in the Results. We did a sensitivity analysis to assess whether there was an interaction between social desirability and randomisation assignment, and then, if there were no interactions, whether the social desirability score was associated with dietary outcomes.

For physical activity outcomes, we used a similar approach as for the dietary outcomes, with the exception that the initial set of predictors also included an indicator variable for day type (ie, weekend *vs* weekday) and interactions between day type, measurement time, and randomisation assignment. After examining the use of wave as a predictor, we did a series of planned hypothesis tests to examine interactions with day type, and then tested the time-by-intervention interaction in the best fitting model.

We did modelling and hypothesis testing similarly for BMI and waist circumference as for the dietary and physical activity outcomes, and additionally adjusted for age and sex.

We had several preplanned secondary outcome analyses. We assessed secondary outcomes at an α level of 0·05. We calculated difference scores, T2 minus T1 and T3 minus T1, for the secondary outcomes of perceived stress and anxiety. We fit separate general linear mixed models for the T2 minus T1 and T3 minus T1 difference scores to test if the difference score differed by group assignment. Our models controlled for the baseline value and the interaction between baseline value and randomisation assignment. Under the assumption that participants were exchangeable within waiting lists, a random intercept for each garden waiting list produced a compound symmetric variance structure. We used a two degrees-of-freedom test of equality of both intercepts and slopes to test if there was a difference between intervention and control at the α level of 0·05.

We did not have an a priori hypothesis about differences at T3, but we present results for T3 for completeness.

We did all calculations using General Linear Mixed Model Power and Sample Size (known as GLIMMPSE) software (version 3.0).^18^

### Role of the funding source

The funders of the study had no role in the study design, data collection, data analysis, data interpretation, or writing of the report.

## Results

Between Jan 1, 2017, and June 15, 2019, 493 individuals were screened, of whom 291 (59%) completed baseline measurements and were randomly assigned to either a garden plot (intervention group; n=145) or the waiting list (control group; n=146; figure). 237 (81%) of the 291 participants attended a health visit with study staff and completed a health survey at all three timepoints for wave 1 (n=50; n=24 in the intervention group and n=26 in the control group) and wave 2 (n=97; n=47 in the intervention group and n=50 in the control group), and the first two timepoints in wave 3 (n=90; n=44 in the intervention group and n=46 in the control group). The number of participants who contributed data for each outcome at each timepoint (ie, T1 and T2 or T3, or both) in the intervention and control groups is shown in the figure. Differing numbers by timepoint, outcome measure, and randomisation assignment reflect loss to follow-up or participant refusal to complete a specific outcome measure at a specific timepoint. There was no differential missingness by group assignment, sex, age, or income (all p>0·05; appendix p 2). Median time from enrolment to T2 was 154 days (IQR 139–180) and T3 was 312 days (290–348).

36 (25%) of 145 participants in the intervention group and 27 (18%) of 146 in the control group were lost to follow-up. During the study, one (1%) participant in the intervention group had an intervention-attributable adverse event: an allergic reaction in the garden. 13 (9%) of 145 participants assigned to the intervention decided not to garden after randomisation and before gardening began and were lost to follow-up after contributing some data at T1. Four (3%) of 146 participants in the control group refused to remain on the waiting list and began to garden; two (50%) remained in the study and contributed data during follow-up, and two (50%) left the study after T1 and did not contribute subsequent data. All participant data were analysed as randomised.

Participants were selected from waiting lists for 37 gardens, and there was a different waiting list for each garden for each year, yielding 65 waiting lists. The mean number of participants per garden waiting list was 4·5 (SD 1·9; range 2–12). The mean number of participants randomly assigned to a garden from each waiting list was 2·2 (SD 1·0), with 2·2 (1·0) randomly assigned to the control group. Mean age of participants was 41·5 years (SD 13·5), 238 (82%) of 291 were female, 52 (18%) were male, 99 (34%) were Hispanic, and 18 (13%) of 141 who provided data reported Spanish to be their first language (table 1). Although all participants had not gardened within the 2 years before the trial, 182 (65%) of 281 with available data reported some gardening experience (table 1). Baseline characteristics of the population were not significantly different between the intervention and control groups in terms of age, sex, self-identified ethnicity, household income, BMI, education, primary language, years of gardening experience, smoking, and social desirability score (table 1).

The time-by-intervention interaction was significant for fibre intake (p=0·034; table 2), with differences being non-significantly higher in the intervention group than in the control group at T2 (mean intake: intervention group 21·48 g per day [SE 0·91]; control group 20·07 g per day [0·87]; between-group difference 1·41 g per day [SE 1·24; 99·5% CI –2·09 to 4·92; p=0·25]). There were no significant time-by-intervention effects for the outcomes of combined fruit and vegetable intake and Healthy Eating Index (all p>0·04; table 2). For combined fruit and vegetable intake, the intervention group had a higher mean intake than the control group at T2 but the difference was not significant (mean intake: intervention group 4·96 servings per day [SE 0·26]; control group 4·49 servings per day [SE 0·24]; between-group difference 0·46 servings per day [SE 0·37; 99·5% CI −0·58 to 1·51; p=0·21]). In a sensitivity analysis, we found no time-by-intervention-by-social-desirability effect for any diet outcome (all p>0·05; data not shown).

We found a significant time-by-intervention effect for moderate-to-vigorous physical activity (p=0·012; table 2), but it was not higher in the intervention group than in the control group at T2 (mean activity: intervention group 54·92 min per day [SE 2·62]; control group 49·12 min per day [SE 2·58]; between-group difference 5·80 min per day [SE 3·62; 99·5% CI −4·44 to 16·05; p=0·11]). Moderate-to-vigorous physical activity did not differ between weekend and weekday (data not shown). We found no time-by-intervention effect on sedentary time (p=0·47; table 2). We found no significant time-by-intervention interaction effect on BMI (p=0·99) or waist circumference (p=0·31) with the gardening intervention versus the control group (table 3)

Participants in the intervention group showed greater reductions than the control group in both perceived stress and anxiety between T1 and T2 (table 4; appendix p 3). A higher magnitude of reduction was estimated (on the basis of the model) for participants who were more stressed or anxious at baseline (PSS-10 estimated change from T1: intervention group −3·14 [SE 0·60]; control group −1·12 [0·56] for participants who had a score at the 75th percentile at baseline; GAD-7 estimated change from T1: intervention group −2·15 [0·38]; control group −0·89 [0·39] for participants who had a score at the 75th percentile at baseline).

## Discussion

To our knowledge, this is the first randomised controlled trial of community gardening in a population that was diverse in terms of age, ethnicity, and socioeconomic status. In this study, we found that participation in community gardening in Denver significantly changed the pattern of response over time for the primary outcomes of fibre intake and moderate-to-vigorous physical activity. Additionally, randomisation to community gardening reduced the secondary outcomes of perceived stress and anxiety, with greater reductions in those who started the trial with higher stress or anxiety. These key behaviours and experiences of stress and anxiety are pertinent to the prevention of cancer and other chronic diseases.^2,19^ We found no significant time-by-intervention interaction effects on the primary outcomes of combined fruit and vegetable intake, Healthy Eating Index, sedentary time, BMI, and waist circumference.

Dietary fibre and whole-plant food intake are increasingly recognised as vital health indicators because of clear associations with improved metabolic health, healthy gut microflora, and decreased inflammation.^20^ Fibre is one measure of cumulative exposure to plant foods (ie, vegetables, fruits, legumes, nuts, and whole grains). The estimated mean fibre intake for US adults is 15·9 g per day, which is much lower than the recommended intake of at least 25–38 g per day.^21^ In our study, participants reported mean fibre intake at baseline (intervention group: mean 22·02 g per day; control group: 22·42 g per day) that was higher, on average, than the US population but still below the levels recommended by US health authorities.^21^ The difference between the intervention group and the control group at T2 was 1·41 g per day. In other trials in healthy adults,^22^ the pooled effect of interventions to promote healthy diet on the consumption of dietary fibre was estimated to be 1·97 g per day (range 0·43 to 3·52), placing the results of our trial well within the bounds of other reported values.

Fruits and vegetables are individual components of fibre intake. A systematic review of 34 behaviour-based interventions designed to increase fruit and vegetable intake found that mean increases in total fruit and vegetable intake in studies of adults ranged from a low of 0·29 servings per day to a high of 2·74 servings per day, and that mean increases attributed to interventions were 1·13 servings.^23^ In our study, the difference between the intervention and control groups for combined fruit and vegetable intake at T2 was 0·46 servings per day, placing our intervention within the range of other interventions designed to increase fruit and vegetable intake, although no direct dietary intervention was conducted, with the intervention only providing access and support for gardening. In a previous qualitative study, gardeners reported enjoying the greater accessibility to fruit and vegetables, superior taste and freshness of garden produce, an emotional connection to the food they had grown themselves, the pleasure of eating garden produce with others, and not wanting to waste food as reasons they ate food from the garden.^24^ In future analyses, we will seek to understand more specific changes in dietary intake that occurred from T1 to T2 and T3. The use of multiple days of 24-h recall (ie, 3 days at each of the three timepoints during the trial) will allow us to focus on specific categories of foods and relate behaviour changes to qualitative data that were collected during the trial.

Within the physical activity domain, we found that although there was no significant difference between the two groups at T2, participants in the intervention group did approximately 5·8 min more of moderate-to-vigorous physical activity per day than participants in the control group at this timepoint. In a recent systematic review of physical activity intervention trials that assessed physical activity changes using device-based measures (eg, accelerometry), the authors found that for the 11 trials that included the measure of moderate-to-vigorous physical activity per day, the median baseline measure was 24·7 min per day (range: 4·3–46·3), with participants in the intervention groups completing 10·3 min per day more moderate-to-vigorous physical activity than comparison groups at last follow-up.^25^ Moderate-to-vigorous physical activity is strongly associated with health and wellbeing.^26^ In a systematic review of physical activity and cancer prevention and survival, high levels of physical activity were associated with reduced risk and improved survival among patients with several different cancers; although the exact intensities of physical activity associated with given levels of effect were not detailed.^27^ 2020 WHO guidelines recommend 150–300 min per week of moderate intensity activity to prevent negative health consequences.^28^ Unfortunately, only 27·5% of the global population meets this recommendation.^28^ In our study, community gardening might have increased moderate-to-vigorous physical activity because gardening is not perceived as exercise, but as something fun and useful to do. Previous qualitative research found that gardeners described the process of gardening as fulfilling and pleasing.^29^

In CAPS, participants randomly assigned to the gardening intervention had a larger decrease in the secondary outcomes of perceived stress and anxiety difference scores from T2 to T1, controlling for the baseline level, than those in the control group. Qualitative and observational studies of gardens across different cultural, geographical, and economic contexts have shown that participating in garden-based activity can reduce feelings of stress and anxiety^30^ and our findings align with related research investigating the associations between nature exposure and stress reduction.^31^ Our trial builds on these earlier studies by showing that community gardens can improve the psychosocial experience of diverse populations in the urban context, with increased therapeutic effects for individuals who start with higher levels of stress and anxiety.

Our results suggest that community gardening, as an example of a multicomponent intervention, could be beneficial in changing some key risk factors for cancer and other chronic diseases, thus warranting further investigation. Data from our qualitative interviews and our process assessment will be examined to contextualise the quantitative results and understand how the trial affected study participants.

We included a broad range of outcomes in our study, which was necessary to measure the pleiotropic effects of community gardening. Multiple outcomes require multiple statistical tests, and multiple statistical tests increase the chance of an inflated type 1 error rate, and reduced replicability. In scientific research, researchers usually correct for multiple comparisons within each manuscript separately, and not over many papers based on data from a single trial. The rationale is that each report represents a separate experiment. A trial with multiple outcomes raises similar questions. The choice of whether or not to use a Bonferroni correction so that the entire trial has a total type 1 error rate of 0·05, or to use a total type 1 error rate of 0·05 per outcome is unclear. We chose the second approach, which controlled the type 1 error rate within each outcome, but did not correct for the multiple outcomes, because each outcome was a separate hypothesis.

Strengths of our study include the randomised controlled design that was adequately powered to test hypotheses, the use of robust measurements for diet, and device-based measurements of physical activity duration. Both diet and physical activity were measured over multiple days at T1, T2 and T3, including three 24-h random diet recalls and 7-day thigh-mounted accelerometry. The inclusion of a diverse population and a high rate of completion of measurements (81%) provide a strong basis for generalisation of our findings. Another strength was the use of longitudinal repeated measures to test the time-byintervention hypothesis, which assessed the difference in the patterns of means across time for the intervention and control groups. This hypothesis test is often more powerful than the test of the main effect of an intervention alone. The analytical plan for the trial accounted for possible correlation within waiting lists and gardens, and repeated measures within individuals. Our analysis accounted for social desirability at baseline and assessed possible social desirability bias in the estimation of intervention effect. The groups were not different at baseline in social desirability, and sensitivity analysis showed no difference in intervention effect by social desirability score, which is known to be a source of bias in dietary self-reporting.^32^

Our study had several limitations. One limitation was the exclusion of data from wave 3 at T3 to account for the COVID-19 pandemic. The data were excluded because pandemic-related closures probably would have affected the primary and secondary outcomes and were excluded before unblinding and before any analysis had occurred. The effect of excluding the last timepoint of data resulted in a decreased sample size and thus might have diminished statistical power or attenuated results towards the null. By design, the trial did not have the power to assess intervention effects within subgroups stratified by race, ethnicity, sex, sexual orientation, and socioeconomic status. Diet, stress, and anxiety outcomes were self-reported, which might lead to information bias, although there was no evidence of social desirability bias for dietary reporting in a sensitivity analysis. Finally, a small number of participants refused their assignment or discontinued participation, and there might be other participants who did not report their status to the study staff. Crossover of participants between groups would only bias the results towards the null. Finally, this trial captured the community gardening experience only over 1 year. Whether the effects of intervention will be maintained beyond 1 year is unknown. Because of the time required to establish new activities such as gardening and maintain changes in health behaviours and health status, long-term follow-up would be useful to understand if and how gardeners maintain the changes they adopted in the first year of gardening.

This randomised controlled trial strengthens evidence for community gardening as a comprehensive multicomponent nature-based social intervention that can improve some health behaviours and reduce perceived stress and anxiety in a diverse urban population. Both are important for the prevention of chronic diseases and mental health disorders. Gardening is a nature-based solution that fits within the broader context of urban agriculture systems. A community garden is a setting that could be within reach for citizens across the world and can be tailored to meet the needs of people across different social and economic groups, cultures, geographies, and local environments. Land planners, health officials, and policy makers together can integrate gardens into the fabric of communities, recognise gardens as a primary and permanent natural space, similar to playgrounds, farmers’ markets, bicycle lanes, and public plazas, and invest in programming that supports gardeners across the lifespan.

## Supplementary Material

1

## Figures and Tables

**Figure:**
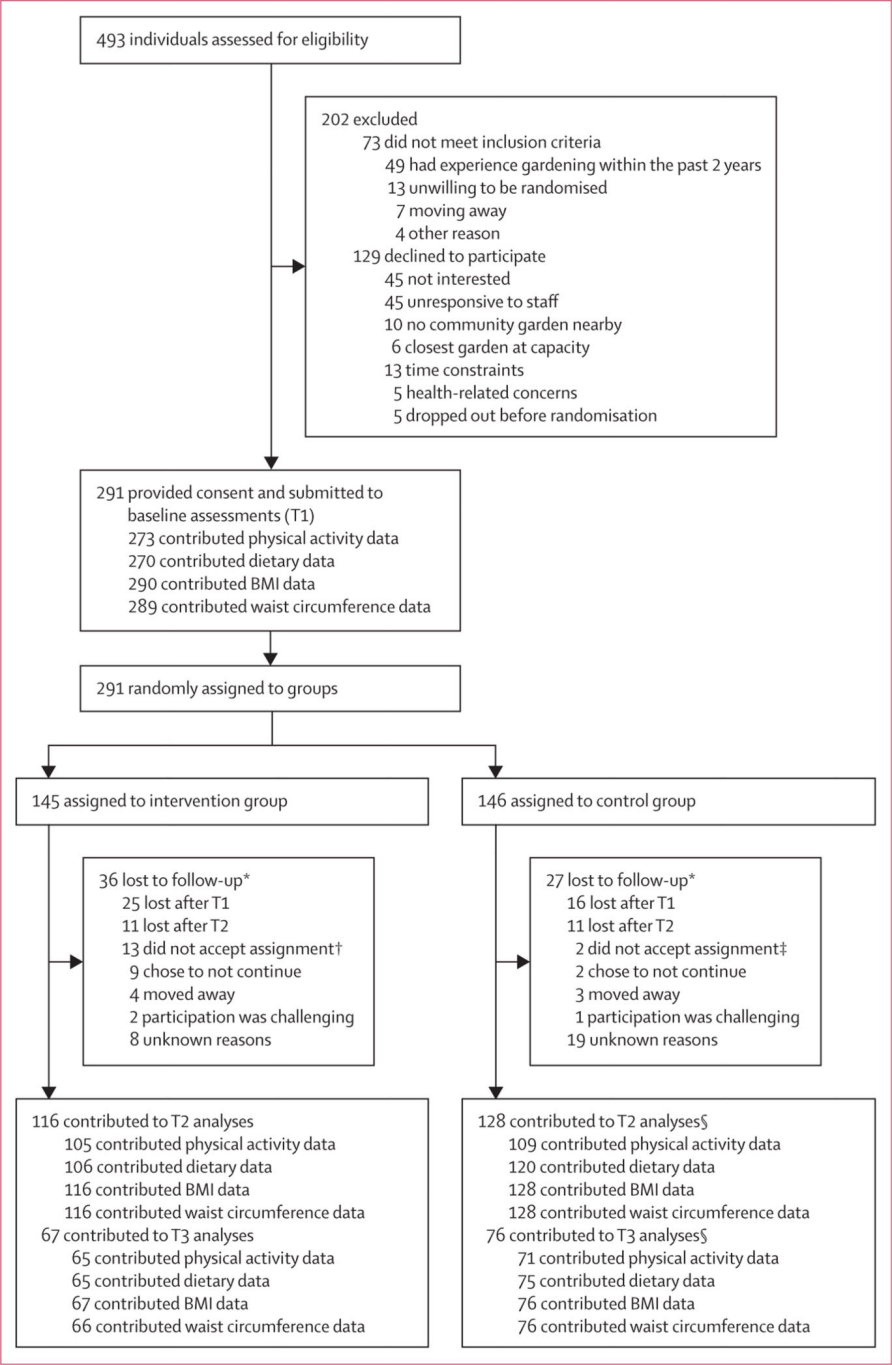
Trial profile The numbers included for analysis might differ for each outcome at each timepoint because of participant refusal to complete specific primary outcome measures, even if they completed other primary outcome measures. Because the COVID-19 pandemic affected data collection and might have had an effect on health behaviours, the study team agreed to exclude wave 3 T3 data from the analysis. T1=timepoint 1. T2=timepoint 2. T3=timepoint 3. *Numbers add up to more than total to include reasons and timepoint when lost to follow-up. †Contributed data at T1 but not T2 or T3; reasons included too busy to participate (n=3), overwhelmed by idea of garden (n=3), health concerns (n=2), did not accept randomised assignment (n=2), moved away (n=1), had unanticipated travel (n=1), and did do not pass garden background check (n=1). ‡Decided to garden after randomisation (ie, crossed over) and left the study after T1 and did not contribute subsequent data; all data were analysed as randomised. §Includes two participants who decided to garden after randomisation (crossed over) and remained in the study and contributed data during follow-up (ie, at both T2 and T3); they were analysed as randomised.

**Table 1:** Baseline characteristics

	Control group (n=146)	Intervention group (gardening; n=145)	p value*
Age, years	41 (14)	42 (13)	0·54
Sex	··	··	··
Female	122 (84%)	116 (80%)	0·53
Male	24 (16%)	28 (19%)	··
Other	-	1 (1%)	··
Self-identified ethnicity	··	··	0·94
Hispanic	51/145 (35%)	48 (33%)	··
Non-Hispanic	94/145 (65%)	97 (67%)	··
BMI, kg/m^2^	28·3 (7·65)	28·2 (7·14)	0·86
Household income, US$	··	··	0·33
<25 000	32/144 (22%)	40/143 (28%)	··
25 000–49 999	47/144 (33%)	48/143 (33%)	··
50 000–74 999	30/144 (21%)	20/143 (14%)	··
≥75 000	35/144 (24%)	35/143 (24%)	··
Education	··	··	0·27
Not a college graduate	55/145 (38%)	47 (32%)	··
College graduate	90/145 (62%)	98 (68%)	··
Primary language	··	··	0·93
English	123/141 (87%)	126 (87%)	··
Spanish	18/141 (13%)	19 (13%)	··
Previous gardening experience, years	··	··	0·10
None	53/142 (37%)	46/139 (33%)	··
<1	19/142 (13%)	37/139 (27%)	··
1–2	29/142 (20%)	19/139 (14%)	··
3–5	15/142 (11%)	19/139 (14%)	··
5–10	12/142 (8%)	6/139 (4%)	··
>10	14/142 (10%)	12/139 (9%)	··
Smoking status			
Smoke every day or some days	20 (14%)	13 (9%)	0·17
Do not smoke not at all	126 (86%)	132 (91%)	··
Social desirability score	6·6 (2·2)	6·7 (2·2)	0·82 score

Data are mean (SD), n (%), or n/N (%).

* For categorical variables, we used Rao-Scott χ^2^ tests to compare study groups, while accounting for clustering among garden waiting lists. A general linear mixed model, using a random effect to control for clustering within garden waiting lists, was used to test for differences in age and social desirability between study groups.

**Table 2:** Time-by-intervention results for diet and physical activity outcomes

	Timepoint 1		Timepoint 2		Timepoint 3		p value
	n	Mean (SE)	n	Mean (SE)	n	Mean (SE)	
**Fruit and vegetable intake, servings per day**							
Intervention (gardening) group	135	4·92 (0·24)	106	4·96 (0·26)	65	4·78 (0·28)	0·28
Control group	135	4·97 (0·23)	120	4·49 (0·24)	75	4·23 (0·26)	··
**Healthy Eating Index**							
Intervention (gardening) group	135	62·9 (0·93)	106	61·7 (1·13)	65	62·3 (1·35)	0·39
Control group	135	62·5 (0·92)	120	59·8 (1·07)	75	60·1 (1·27)	··
**Fibre, g per day**							
Intervention (gardening) group	135	22·02 (0·95)	106	21·48 (0·91)	65	23·21 (1·17)	0·034
Control group	135	22·42 (0·94)	120	20·07 (0·87)	75	19·67 (1·10)	··
**Moderate-to-vigorous physical activity, min per day**							
Intervention (gardening) group	136	52·83 (2·78)	105	54·92 (2·62)	65	45·41 (2·65)	0·012
Control group	137	54·86 (2·75)	109	49·12 (2·58)	71	43·22 (2·57)	··
**Sedentary time, min per day**							
Intervention (gardening) group	136	171·8 (11·5)	105	180·7 (11·3)	65	187·7 (19·0)	0·47
Control group	137	172·7 (11·3)	109	169·7 (11·2)	71	178·6 (18·5)	··

**Table 3:** Time-by-intervention results for adiposity and anthropometric results, controlling for sex

	Timepoint 1		Timepoint 2		Timepoint 3		p value
	n	Mean (SE)	n	Mean (SE)	n	Mean (SE)	
**BMI, kg/m^2^**							
Intervention (gardening) group							
Female	116	28·3 (0·7)	93	28·2 (0·7)	53	28·3 (0·7)	0·99
Male	28	26·1 (1·4)	23	26·1 (1·4)	14	26·2 (1·4)	··
Control group							
Female	122	28·6 (0·7)	107	28·4 (0·7)	64	28·3 (0·7)	··
Male	24	25·1 (1·5)	21	24·9 (1·5)	12	24·9 (1·5)	··
**Waist circumference, cm**							
Intervention (gardening) group							
Female	116	90·5 (1·6)	93	91·3 (1·7)	53	91·1 (1·7)	0·31
Male	28	93·6 (3·2)	23	93·6 (3·3)	13	94·4 (3·4)	··
Control group							
Female	121	91·3 (1·6)	107	91·9 (1·6)	64	92·1 (1·6)	··
Male	24	93·5 (3·4)	21	91·2 (3·5)	12	91·3 (3·6)	··

**Table 4:** Difference score model results for perceived stress (PSS-10) and generalised anxiety (GAD7)

	Timepoint 2 vs timepoint 1					Timepoint 3 vs timepoint 1				
	n	25th percentile	50th percentile	75th percentile	p value	n	25th percentile	50th percentile	75th percentile	p value
**PSS-10**										
Baseline value for all study participants	··	10	14	19	··	··	10	14	19	··
Intervention (gardening) group	114	0·46 (0·61)	−1·14 (0·50)	−3·14 (0·60)	0·025	71	1·30 (0·87)	−0·68 (0·68)	–3·16 (0·85)	0·58
Control group	124	1·66 (0·58)	0·42 (0·48)	–1·12 (0·56)	··	74	1·44 (0·84)	−0·08 (0·67)	–1·97 (0·79)	··
**GAD-7**										
Baseline value for all study participants	··	2	5	8	··	··	2	5	8	··
Intervention (gardening) group	117	0·67 (0·42)	−0·74 (0·33)	−2·15 (0·38)	0·044	70	0·79 (0·58)	−0·47 (0·47)	−1·74 (0·52)	0·49
Control group	126	1·49 (0·40)	0·30 (0·32)	−0·89 (0·39)	···	75	1·26 (0·57)	0·15(0·46)	−0·95 (0·52)	··

The hypothesis is of no intervention effect on difference score, controlling for baseline values. Mean (SE) of the respective stress and anxiety scores at baseline are preported by percentile. GAD-7=General Anxiety Disorder 7. PSS-10=Perceived Stress Scale 10.

## Research in context

### Evidence before this study

On March 1, 2022, we searched MEDLINE, PubMed, PsychINFO, and Advanced Google Search, with no date or language restrictions, using the terms “garden”, “gardening”, AND “randomised”. Our search identified mostly systematic reviews and non-randomised studies on the associations between garden participation and a range of health behaviours and outcomes including diet and activity, BMI, and health status, with most studies showing positive evidence for garden interventions as a health promotion strategy. Three studies were randomised controlled trials of gardening among adults in home settings, but none involved community gardens.

### Added value of this study

To our knowledge, the CAPS trial is the first randomised controlled trial to study the effect of community gardening on diet, physical activity, and psychosocial outcomes in urban adults who are diverse in terms of age, ethnicity, and socioeconomic status. We found that a multicomponent, community-based intervention increased fibre intake and moderate-to-vigorous physical activity and reduced perceived stress and anxiety, but did not significantly change anthropometric outcomes, healthy eating index, combined fruit and vegetable intake, or sedentary time.

### Implications of all the available evidence

Community gardens provide a theory-informed, non-medical, community-based, and nature-based opportunity to improve chronic disease risk factors including diet, physical activity, and stress and anxiety adults who are diverse in terms of age, ethnicity, and socioeconomic status. Evidence indicates that garden interventions are acceptable to diverse populations across different age groups and are feasible. Community garden interventions can be considered a part of community-based strategies, including nature-based social prescribing, to reduce risk factors for cancer and chronic disease, and more broadly, address health and wellbeing.
