# Supplementary material for: Effects of a community gardening intervention on diet, physical activity, and anthropometry outcomes in the USA (CAPS): an observer-blind, randomised controlled trial
Source: Lancet Planet Health. Author manuscript; Available in PMC 2023 Feb 17. (PMC9936951; doi:10.1016/S2542-5196(22)00303-5)
Supplement: 1 [file NIHMS1862865-supplement-1.pdf]

THE LANCET
Planetary Health

Supplementary appendix

This appendix formed part of the original submission and has been peer reviewed. We post it as supplied by the authors.

Supplement to: Litt JS, Alaimo K, Harrall KK, et al. Effects of a community gardening intervention on diet, physical activity, and anthropometry outcomes in the USA (CAPS): an observer-blind, randomised controlled trial. *Lancet Planet Health* 2023; **7:** e23–32.

Supplementary Materials Table of Contents

Supplementary Table A…………………………………………………………………………… 2

Supplementary Figure A………………………………………………………………………….. 3

CONSORT Checklist…………………………………………………………………………… 4

UCB IRB Human Subjects Protocol………………………………………………………………… 6

Supplementary Table A: Missingness, overall, and by randomization group

		Entire cohort (N = 291)		
		**Completed all measurements**	**Missed at least one measurement**	**p-value**
**Randomisation Allocation**				
	Garden	115	30	0·56
	Control	122	24	
**Sex**				
	Male	41	11	0·55
	Female	196	42	
**Age**				
	18-30	51	12	0·99
	31-40	78	16	
	41-50	39	9	
	51-60	42	9	
	61+	27	5	
**Income**				
	<$25,000	59	13	0·56
	$25,001-$50,000	80	15	
	>$50,001	94	26	

* Because the SARS-CoV-2 pandemic affected data collection and might have had an impact on health behaviours, the study team agreed to exclude Wave 3, T3 data from the analysis. The decision was made a priori, while the blind was still in force. A participant in Wave 1 or 2 was considered to have completed all measurements if they completed at least one primary outcome measurement at T1, T2 and T3. A participant in Wave 3 was considered to have completed all measurements if they completed at least one primary outcome measurement at T1 and T2.

Supplementary Figure A: Predicted differences of perceived stress (PSS-10) and generalized anxiety (GAD7) by scores measured at baseline: a) difference in PSS-10 at T2, b) difference in PSS-10 at T3, c) difference in GAD7 at T2 and d) difference in GAD7 at T3

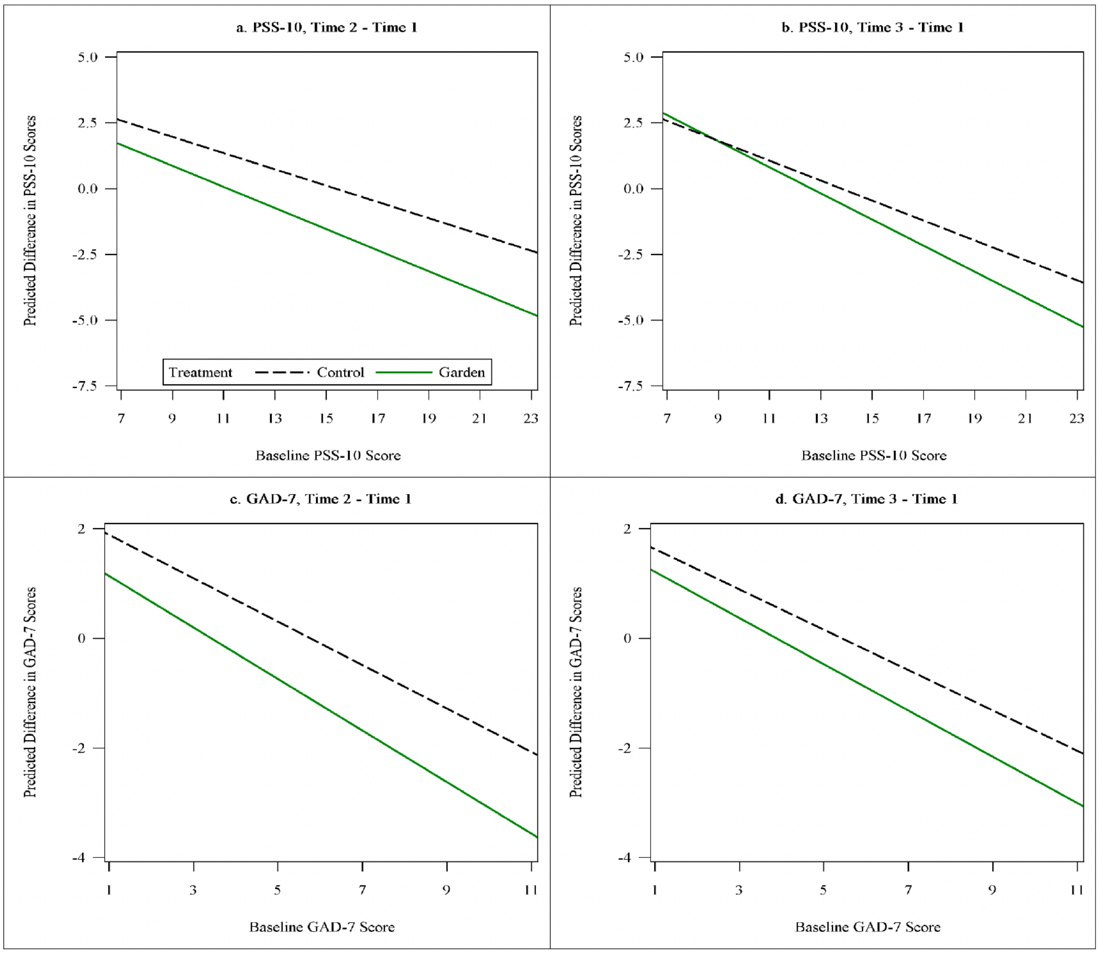

Footnote: Panel A is PSS-10, T2 –T1, Panel B is PSS-10, T3-T1, Panel C is GAD 7, T2 – T1, Panel D is GAD 7, T3 – T1.

CONSORT 2010 checklist of information to include when reporting a randomised trial*

Section/Topic	Item No	Checklist item	Reported on page No
**Title and abstract**			
	1a	Identification as a randomised trial in the title	1
	1b	Structured summary of trial design, methods, results, and conclusions (for specific guidance see CONSORT for abstracts)	Abstract (p.4)
**Introduction**			
Background and objectives	2a	Scientific background and explanation of rationale	5-6
	2b	Specific objectives or hypotheses	5
**Methods**			
Trial design	3a	Description of trial design (such as parallel, factorial) including allocation ratio	Abstract; p.7
	3b	Important changes to methods after trial commencement (such as eligibility criteria), with reasons	7
Participants	4a	Eligibility criteria for participants	Abstract; p.7
	4b	Settings and locations where the data were collected	Abstract; 6-7
Interventions	5	The interventions for each group with sufficient details to allow replication, including how and when they were actually administered	7
Outcomes	6a	Completely defined pre-specified primary and secondary outcome measures, including how and when they were assessed	Abstract; pp. 8-9
	6b	Any changes to trial outcomes after the trial commenced, with reasons	9
Sample size	7a	How sample size was determined	8-9
	7b	When applicable, explanation of any interim analyses and stopping guidelines	NA
Randomisation:			
Sequence generation	8a	Method used to generate the random allocation sequence	7
	8b	Type of randomisation; details of any restriction (such as blocking and block size)	Abstract; p. 7
Allocation concealment mechanism	9	Mechanism used to implement the random allocation sequence (such as sequentially numbered containers), describing any steps taken to conceal the sequence until interventions were assigned	7
Implementation	10	Who generated the random allocation sequence, who enrolled participants, and who assigned participants to interventions	7
Blinding	11a	If done, who was blinded after assignment to interventions (for example, participants, care providers, those	Abstract; p. 7

		assessing outcomes) and how	
	11b	If relevant, description of the similarity of interventions	NA
Statistical methods	12a	Statistical methods used to compare groups for primary and secondary outcomes	Abstract; 8-9
	12b	Methods for additional analyses, such as subgroup analyses and adjusted analyses	9-10
**Results**			
Participant flow (a diagram is strongly recommended)	13a	For each group, the numbers of participants who were randomly assigned, received intended treatment, and were analysed for the primary outcome	10
	13b	For each group, losses and exclusions after randomisation, together with reasons	10
Recruitment	14a	Dates defining the periods of recruitment and follow-up	6; 10
	14b	Why the trial ended or was stopped	NA
Baseline data	15	A table showing baseline demographic and clinical characteristics for each group	Table 1
Numbers analysed	16	For each group, number of participants (denominator) included in each analysis and whether the analysis was by original assigned groups	Figure 1, P.10
Outcomes and estimation	17a	For each primary and secondary outcome, results for each group, and the estimated effect size and its precision (such as 95% confidence interval)	Abstract; p. 11
	17b	For binary outcomes, presentation of both absolute and relative effect sizes is recommended	
Ancillary analyses	18	Results of any other analyses performed, including subgroup analyses and adjusted analyses, distinguishing pre-specified from exploratory	11
Harms	19	All important harms or unintended effects in each group (for specific guidance see CONSORT for harms)	10
**Discussion**			
Limitations	20	Trial limitations, addressing sources of potential bias, imprecision, and, if relevant, multiplicity of analyses	13-14
Generalisability	21	Generalisability (external validity, applicability) of the trial findings	13
Interpretation	22	Interpretation consistent with results, balancing benefits and harms, and considering other relevant evidence	11-14
**Other information**			
Registration	23	Registration number and name of trial registry	Abstract; p.7
Protocol	24	Where the full trial protocol can be accessed, if available	7
Funding	25	Sources of funding and other support (such as supply of drugs), role of funders	Abstract; p.15

*We strongly recommend reading this statement in conjunction with the CONSORT 2010 Explanation and Elaboration for important clarifications on all the items. If relevant, we also recommend reading CONSORT extensions for cluster randomised trials, non-inferiority and equivalence trials, non-pharmacological treatments, herbal interventions, and pragmatic trials. Additional extensions are forthcoming: for those and for up to date references relevant to this checklist, see www.consort-statement.org.

University of Colorado Boulder Institutional Review Board Office

Project Title:
Community Activation for Prevention (CAPs): A Randomized Controlled Trial of Community Gardening

Protocol #: **16-0644**
Protocol Version: 7

Principal Investigator: Jill S. Litt, PhD
PI Address: Sustainability, Energy, & Environment Complex (MacAllister)
4001 Discovery Drive, Mail Stop: 397 UCB
Boulder, Colorado 80303
Telephone: 303-735-4519
Date: March 1, 2018

Key Personnel
Jill S. Litt, PhD, Principal Investigator, University of Colorado Boulder
Tessa Crume, PhD, Co-Investigator, Colorado School of Public Health
Deborah Glueck, PhD, Co-Investigator, Colorado School of Public Health
Jenn Leiferman, PhD, Co-Investigator, Colorado School of Public Health
Richard Hamman, MD, DrPH, Colorado School of Public Health
Kaigang Li, PhD, Co-Investigator, Colorado State University

Key Personnel: Non-CU Institutions
Katherine Alaimo, Co-Investigator, Michigan State University
James Hebert, PhD, Co-Investigator, University of South Carolina

Other Collaborators
Michael Buchenau, MLA, Denver Urban Gardens
Tom Gurley, University of South Carolina

Roles and Responsibilities of Investigators and Staff
Dr. Litt is the principal investigator of this newly funded project. She will oversee all aspects of the project including oversight of personnel, training of personnel, protocol development and implementation, data analysis, research dissemination and grant partner coordination and management. **Dr. Glueck** is listed as key personnel because of her instrumental role on the project as a co-developer in this grant concept and proposal. However, her role will be constrained to the design of the statistical analytic framework and the analysis of the study data. She will oversee the design of the study database, the management and analysis of data, and the preparation of manuscripts related to this project. **Dr. Leiferman** will assist in the development and monitoring of the process evaluation and the selection and assessment of social and psychological constructs used in our health survey. Dr. Tessa Crume will assist in the development of the recruitment and data management systems and will advise doctoral students in the development of these data systems using REDcap. **Dr. Kaigang Li** will oversee the assessment of physical activity among study participants. Dr. Li will assist in the training of physical activity monitoring, the development of protocol for monitoring physical activity and the collection and analysis of physical activity data. He will be involved in all aspects of the project and in the preparation of study manuscripts. **Dr. Katherine Alaimo** is a co-Investigator from Michigan State University. Although the American Cancer Society did not allow Dr. Alaimo to be listed as co-PI or allow us to set up a multi-PI structure, she will co-lead this project with me and will oversee the engagement of our study advisors from across the nation. She will work closely with Dr. Leiferman on the process evaluation and will coordinate closely with Dr. Hebert of University of South Carolina on the data collection protocols for diet assessment. She will help manage project personnel, interface with garden leaders and study participants. **Dr. James Hebert** is a co-Investigator from University of South Carolina and will oversee the diet assessment component of the study, in close coordination with Drs. Alaimo and Litt.

Dr. Richard Hamman will serve as a consultant to the project. Specifically, he will provide clinical trial oversight as it relates to the design and monitoring of the trial. **Mr. Tom Hurley** will serve as the staff lead to manage the

collection of random diet recalls. Mr. Hurley is based at the University of South Carolina. Finally, **Mr. Michael Buchenau** is the executive director of Denver Urban Gardens and will provide input in the execution of the trial, the strategies to engage garden leaders, to address issues as they arise with garden engagement and recruitment of study participants. Mr. Buchenau will not be involved in data collection or have access to study databases. He will serve in a more supervisory role and also support our Advisory Committee led by Dr. Alaimo.

I. Objectives and Specific Aims

We propose a randomized controlled trial to determine whether **community gardening** improves **cancer-preventive behaviors** among a **multi-ethnic, low-income adult population** and elucidate the **pathways** that shape cancer-preventive behaviors.(1) A randomized controlled trial is needed to demonstrate that the observed behavioral changes are due to the effect of gardening as an intervention rather than self-selection by gardeners.

Denver Urban Gardens (DUG) randomly assigns people on their wait lists to garden plots or further waiting, thus creating conditions and precedent to conduct our study. As part of the study, we will recruit study participants from garden wait lists. Once waitlists are established, we will screen all waitlisters for their interest in the study and their eligibility to participate. Eligibility is determined by their age (18 years or older) and their past gardening experience (cannot have gardened in the past 2 years). From the waitlist and our screening process, two groups of waitlisters will emerge. One that includes folks not interested or eligible to participate (Group 1) and the other that includes waitlisters that would like to participate in the study and have provided written consent to participate (Group 2).

- Group 1 will have access to garden plots allocated by garden leaders and this group of wait listers will be randomly assigned to garden or wait-list based on the availability of plots for this group (this availability will vary by garden).
- Group 2 will have access to another set of garden plots allocated to the study by garden leaders (this availability will vary by garden). Based on availability of garden plots and sufficient interest in the study by waitlisters at a particular garden, DUG will randomize the second group to garden or wait.

Availability of plots: DUG will build 10 new gardens per year for 2017-2019. We anticipate ~40 applicants for each new garden with only ~25 plots available (40 applicants x 10 gardens = 400 applicants per year). Moreover, there are 100 existing gardens eligible for our study, of which 90% have a waitlist. Assuming 4-6 new families join an existing garden waitlist (personal communication, DUG staff), we will recruit from an additional pool of ~360-540 applicants per year (for a total pool of 760-940 applicants per year)**.** Thus, we will be able to recruit the 104 participants per year for 3 years (non-gardeners prior to the study) needed to have strong power, even with 30% loss to follow-up. In total, this study will enroll 156 gardeners and 156 non-gardeners over 3 years. At baseline (T1: pre-gardening/March), harvest time (T2: August), and post-intervention (T3: March), we will collect diet, sedentary time, physical activity, and anthropometric data.

Importantly, the population for our trial mirrors populations of interest for cancer prevention efforts. Socioeconomic and racial/ethnic inequities in cancer outcomes are persistent in Colorado and may worsen because of rising poverty, inequality in income and social exclusions in communities of color and low income neighborhoods.(2) In a population-based survey of Denver residents, more than 50% of community gardeners were female, more than 30% self-identified as Hispanic or African-American, and almost 50% reported household income less than $50,000.(3) Proof of the hypotheses of this trial will lead to the conclusion that gardens are a scalable, sustainable, and feasible intervention that encourages people to make healthy lifestyle choices that reduce their cancer risk. Consistent with the ACS Stay Healthy Initiative, our study aims to:

***Aim 1:* Determine whether community gardening leads to increased intake of fruits and vegetables and thus increased intake of fiber, lower total energy intake, and higher Healthy Eating Indices**.

***Aim 2:* Determine whether community gardening leads to reduced sedentary time, increased physical activity (including light, moderate, and vigorous activity), and reduced age-associated weight gain.**

We hypothesize that the intervention group will improve diet (Aim 1) and activity behaviors (Aim 2), and maintain BMI and waist circumference (Aim 2) over baseline (T1) during the peak gardening season (T2) more than the control group, and that these differences will be sustained (T3).

***Aim 3:* Elucidate the mechanisms underlying the differences found in diet, activity, BMI and waist circumference between gardeners and non-gardeners.** We hypothesize that community gardening influences health behaviors through intrapersonal (e.g., subjective norms), interpersonal (e.g., social support) and environmental (e.g., neighborhood attachment and environmental aesthetics) processes.(4-12)

II. Background and Significance

Cancer remains the second leading cause of death in the U.S., with 1.65 million new cancer cases in 2014.(13, 14) Health care costs attributable to cancer were more than $216 billion in 2009.(14-16) Socioeconomic disadvantage, access to fruits and vegetables, and access to supportive environments for physical activity are critical areas for reducing cancer risk.(17) The American Cancer Society and others consistently report that, in addition to smoking, the *major modifiable risk factors for cancer are diet, including low fruit, vegetable and fiber intake, prolonged sedentary time and physical inactivity*.(13, 18-26) People of color living in communities with higher levels of poverty (>30%) experience neighborhood conditions that can exacerbate their health status.(27) This is due, in part, to exposure to health-compromising conditions such as lack of access to healthy food and outdoor activity spaces.(27) Given the burden of cancer and costs that could be prevented by changes in health behaviors, a primary question in cancer prevention remains: How do we encourage all people to make healthy lifestyle choices that reduce their risk of cancer? **We hypothesize that community gardens – green spaces where individuals from more than one family grow food communally or side-by-side – represent a place-based strategy for eliminating cancer-preventive behavioral health disparities.** In Denver, there are 160 community gardens. The majority of these gardens are located in socioeconomically (see Figure 1), racially (15% African American), and ethnically diverse (32% Hispanic) neighborhoods.(28) Our analysis of socioeconomic conditions in Denver shows that 91% of community gardens reach block groups that exceed the Federal Poverty Threshold (>20% of persons in poverty).

Figure 1: Eligible Gardens and Percent Minority Population in Denver and Aurora, CO

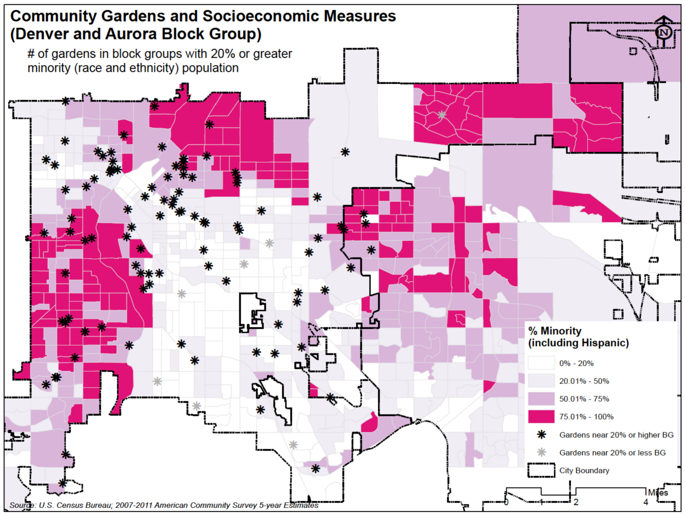

Using cross-sectional studies, Litt, Alaimo and others have shown that community gardeners eat more fruits and vegetables, engage in more leisure and moderate physical activity, and have lower BMI.(4-10, 29, 30) Moreover, gardeners report greater social support and involvement, which are important predictors of cancer-preventive behaviors and self-rated health.(31, 32) Thus, gardens have the potential to reduce the burden of cancer, which account for nearly 25% of U.S. deaths.(33)'(14, 16) Our proposed randomized controlled trial of community gardening takes advantage of a natural experiment to rigorously test our hypothesis, without the self-selection bias that has weakened prior research. This trial will allow us to focus on cancer-preventive behaviors among low income, multi-ethnic populations. **The population for our trial mirrors populations of interest for cancer prevention.** In a survey of Denver residents, over 50% of gardeners were female, more than 30% self-identified as Hispanic or African-American, and 50% reported HH incomes less than $50,000.(3)

Results from our controlled trial will be used **to improve community-level cancer prevention strategies by building and strengthening gardens and thus offering socio-environmental pathways to reduce cancer disparities and improve health equity**. A community garden is more than a place to grow food – it represents **an environmental change with a strong social component**. Both components are crucial to supporting and sustaining health behavior change in a **culturally meaningful** way.(34, 35) Our trial will examine mechanisms for health behaviors and weight maintenance and thus can offer insight into the development of other multi-component behavioral interventions for low-income communities and communities of color.

Statement of Cancer Relevance

According to a recent study, nonsmoking adults whose lifestyles were most consistent with the 2006 American Cancer Society cancer prevention guidelines for weight control, physical activity, diet, and alcohol were significantly less likely to die from cancer.(36) As stated in the most recent *ACS Guidelines on Nutrition and Physical Activity for Cancer Prevention,* (p.31)(13) "community efforts are therefore essential to create an environment that facilitates healthy food choices and physical activity." Synergistic interventions that simultaneously encourage multiple health behaviors and are grounded in the community are needed. In a comprehensive way that goes beyond the limitations of many traditional public health interventions, community gardens provide an aesthetic, social, fun, and low-cost "nudge" that decreases barriers and provides opportunities to be active, grow fruits and vegetables, and form deep social connections. This research will have implications for how health behavior and obesity prevention efforts are

designed and implemented at the community level. If successful, this intervention will reach populations that often lack access to neighborhood-level amenities and services, safe and healthy food, and supportive environments for active living.

III. Preliminary Studies

Our previous published studies show that community gardeners eat fruits and vegetables on more occasions per day (1.8 more times/day, p<.001) and are more likely to avoid age-associated increase in body mass index (BMI) (Denver: p<0.05).(4-7, 32) The increase in fruit and vegetable consumption across all social and demographic groups is almost two times larger than the increase seen across most other published interventions.(37, 38) The mechanistic underpinnings of why gardeners exhibit improved health outcomes remain unclear. Our study aims to determine whether community gardening improves cancer-preventive behaviors among a multi-ethnic, low-income adult population and elucidate the pathways that shape cancer-preventive behaviors.(1)

IV. Research Study Design

Overview

We propose a pilot randomized controlled trial of community gardening, a social-environmental intervention that addresses health behavior change and chronic disease prevention. We will recruit 312 prospective gardeners who are the Denver Urban Gardens wait lists. **DUG randomly assigns people on each garden wait list to available plots, using a lottery. This creates an opportunity to recruit individuals from existing garden waitlists to participate in our study.** Individuals randomized to the garden intervention will receive a standardized garden resource package, which includes the following:

1. A garden plot: **Gardens are predominantly located in low-income communities and communities of color**. Intervention participants will be assigned a plot in a DUG garden.
2. Seeds and plant starts: DUG will provide new gardeners with vegetable seeds and plant starts.
3. Introductory gardening workshop: DUG will offer beginner gardening classes.
4. Social events: New gardeners will be included in garden-based social events, one-to-one and one-to-many garden mentoring, available through DUG's Master Gardener Program.

The non-gardening group will remain on the DUG wait lists and will not receive these resources during the trial.

DUG will build 10 new gardens per year for 2017-2019. We anticipate ~40 applicants for each new garden with only ~25 plots available (40 applicants x 10 gardens = 400 applicants per year). Moreover, there are 100 existing gardens eligible for our study, of which 90% have a waitlist. Assuming 4-6 new families join an existing garden waitlist (personal communication, DUG staff), we will recruit from an additional pool of ~360-540 applicants per year (for a total pool of 760-940 applicants per year). Thus, we will be able to recruit the 104 participants per year for 3 years (non-gardeners prior to the study) needed to have strong power, even with 30% loss to follow-up. In total, this study will enroll 156 gardeners and 156 non-gardeners over 3 years. At baseline (T1: pre-gardening/March), harvest time (T2: August), and post-intervention (T3: March), we will collect diet, sedentary time, physical activity, and anthropometric data.

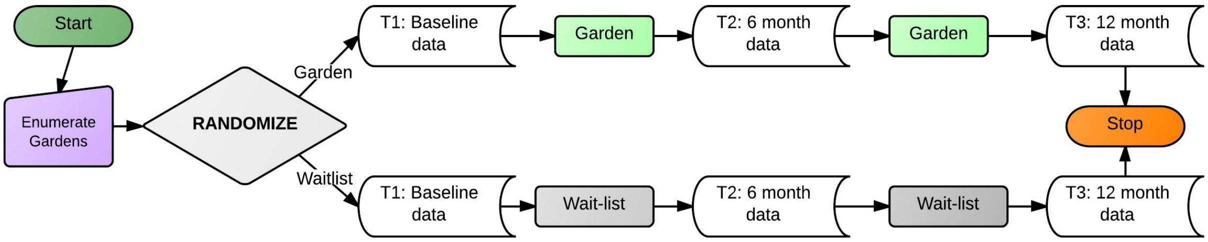

Figure 2: Community Activation for Prevention (CAPs): A Randomized Controlled Trial of Gardening

V. Funding

This study is funded by the American Cancer Society Grant #**130091-RSG-16-169-01-CPPB**.

Table 1: Targeted Enrollment **VI. About the Subjects**

TARGETED/PLANNED ENROLLMENT: Number of Subjects			
**Ethnic Category**	**Sex/Gender**		
	**Females**	**Males**	**Total**
Hispanic or Latino	36	33	69
Not Hispanic or Latino	126	117	243
**Ethnic Category: Total of All Subjects ***			
**Racial Categories**			
American Indian/Alaska Native	2	1	3
Asian	6	3	9
Native Hawaiian or Other Pacific Islander	0	0	0
Black or African American	20	21	41
White	135	124	259
**Racial Categories: Total of All Subjects ***	162	150	312

* The "Ethnic Category: Total of All Subjects" must be equal to the "Racial Categories: Total of All Subjects."

The selection of participants will be based on whether they are 18 years and older, whether they have not gardened for at least 2 years and whether they have completed their application for a plot at a Denver Urban Gardens site. Gardens will be located in predominantly low income and minority neighborhoods and thus we expect that participants will be ethnically and socioeconomically diverse. Special vulnerable populations will not be intentionally recruited for this study (See Table 1 for targeted enrollment for this pilot study). We will work closely with gardens that would like to participate in the study. This desire to participate will be based on garden leader engagement early in the design of this project.

Garden leaders will be briefed on the study and asked whether they would like to participate by reserving garden plots to the study. Garden leaders will be provided a $50 incentive for each garden plot reserved for the study. This incentive has been suggested by Denver Urban Gardens to help support garden activities at the garden. If a garden reserves 4 plots for the study, they will receive $200 directly deposited into a garden-specific spending account. This will cover expenses such as garden plot fee offsets for those who can not afford to pay plot fees, water bills, supplies, and seeds and transplants. Because of the decentralized nature of the gardens in Denver, each garden is required to cover its operation costs, which are only partially covered by plot fees. These incentives will help offset these financial responsibilities. Additionally, we will invite garden leaders, from participating community gardens, and DUG staff to consent to provide feedback through surveys designed to provide information about how the intervention is working, experiences in the garden, DUG and study staff interactions with garden leaders and garden leader interactions with DUG and study staff as well as study participants and opportunities for future collaboration in waves 2 and 3 of the research study. These questions inform the process evaluation. Participation is voluntary and is separate from garden leader decisions to reserve plots in their community garden for the research study.

VII. Vulnerable Populations

This study aims to address opportunities to improve health behaviors and health status among predominantly low income and minority residents in Denver, who face the greatest barriers to safe and beautiful places for activity and healthy food. The community garden provides a context through which we can explore how neighborhood settings can lead to behavioral changes that support healthy and active lifestyles. Because this study is a follow up to a decade of community-academic collaboration between the PI and Denver Urban Gardens, our research questions and approach reflect the input of gardeners and program staff to help guide the study and inform the procedures for study recruitment, implementation and dissemination of research results. Moreover, we will partner with garden leaders to engage with wait-listers and will hire staff from the community to facilitate authentic engagement, communication, and encouragement. Because of the schedule for data collection, we will be closely aligned with study participants and have frequent opportunities to assess participant engagement and satisfaction.

VIII. Recruitment Methods

1. Sampling frame. Study personnel will distribute flyers and door hangers to households and apartment complexes within a 1/4 mile of new gardens, which will be predominantly located in low-income communities and communities of color, announcing the new garden and inviting individuals to submit an application for a garden plot. Additionally, DUG will post new garden information on its website and list serve on behalf of the study. Prospective gardeners will be directed to contact the "New Gardens" DUG coordinator. Current data show that almost all existing DUG gardens have a wait list. Demand for garden plots will likely exceed supply for new gardens (personal communication with DUG staff, January, 2016) but in the case of new gardens, advertisement is necessary to drive individuals to the garden waitlist. This activity not only helps the study but also helps DUG with the launch of new gardens, which can take several years to build interest and sustain participation.

For the purposes of the study, DUG has agreed to add one brief section to the application, which will include information about the trial and will ask prospective gardeners if they would agree to be contacted by study personnel.

2. Study eligibility. Study personnel will contact prospective gardeners who have agreed to be contacted to determine whether the prospective gardeners are study-eligible (see pre-screening consent form). Prospective gardeners will be contacted by telephone or email to arrange a call or in-person visit. We tested this procedure in our pilot trial and found the approach to be successful. All study personnel will be bilingual Spanish/English. Study participants must be: 1) able to give informed consent in English or Spanish; 2) aged 18 or over; 3) currently on the wait list for a new garden; 4) willing to undergo study measurements; and 5) not have gardened in the past 2 gardening seasons. We will record the number and demographics of people who do not meet eligibility requirements.

3. Randomization. After we determine study eligibility, and obtain consent, we will subdivide the wait list for each garden into two lists: consented study participants and non-participants. DUG will conduct two lotteries to assign gardens, one for the study participants, and one for the non-participants. For the randomization, DUG will use balanced block randomization schedules prepared in a blinded fashion by the study biostatistician (Dr. Glueck). All applicants will have an equal chance of obtaining a garden plot. Through previous experience, we estimate that roughly half of people on each wait list will get a plot. We will analyze as we randomize. Even if there is dropout or crossover, we will still analyze under intent to treat.

We will work closely with gardens that would like to participate in the study. This desire to participate will be based on garden leader engagement early in the design of this project. Garden leaders will be briefed on the study and asked whether they would like to participate by dedicating garden plots for the study. Moreover, we will focus our garden engagement in areas of highest need where gardens are less established and more vulnerable to turnover. Thus, gardens with extensive waitlists will most likely not be a part of our study and thus will not face the risk of displacing people who have been waiting several years for plots. In the higher risk areas, we will raise awareness about the garden in surrounding neighborhoods through flyering and door hangers. Our pilot randomized controlled trial in Summer 2016 showed that this method was effective in recruiting individuals to sign up for the waitlist. In the instance of this pilot study, the study led to the successful implementation of a new garden in a very low income area of Denver where low participation threatened the viability of the garden.

List recruitment methods/materials and attach a copy of each in eRA
1. Flyer
2. Newsletter
3. Website and listserve

4. Door Hangers

IX. Compensation

Participants will receive incentives following the completion of each data collection point: a $25 gift card at the first time point, a $50 gift card at the second time point, and a $75 gift card at the last time point. These payments will be made at baseline (T1: pre-gardening/March), harvest time (T2: August), and post-intervention (T3: March). Measurements will include 3 random diet recalls by phone, sedentary time and physical activity using accelerometers and objectively measured height and weight.

These incentives will cover compensation for cell phone usage time. Moreover, once residents complete their applications for a garden plot, they will: 1) be added to the wait list; and 2) access the Underground Blog managed by DUG and announcements about seminars, trainings and community events. DUG events are well attended with over 300 individuals recently attending the 2016 gardener leader symposium.

Participants will also have the opportunity to be entered into a drawing to win one of ten CAPS prizes: 1) five (5) a garden package (5 to 7-piece gardening tool set, garden book, gardening gloves), 2) five (5) $25 gift card to Nick's Garden Center and Farm Market, which is a local community partner of Denver Urban Gardens. One entry will be given per participant for every successful contact point they complete, during Check Point 1 (CP1) and Check Point 2 (CP2), with a maximum of 2 entries per participant. Entries will be associated with the completed survey and stored in the participant REDCap database, and exported for entry at the time of the drawing. Odds of winning are 1 in 10, with chances of winning dependent on the total completed contact point surveys and number of participants per study year.

X. Consent Process

If a prospective gardener meets eligibility requirements, the study personnel will contact them by phone, give a brief description of the study, and ask the prospective gardener if they want to participate in the study. During this process, the intervention and randomization protocol will be explained as well as the data collection procedures and risks and benefits of participating in the study. Informed consent forms will be available both in Spanish and in English and will be reviewed by the University of Colorado Boulder Institutional Review Board. At the first health visit, informed consent will be explained and signed. At the participant's request, a copy will be made available. Visits will be conducted in a private room at Denver Urban Garden's office, our partner site.

Garden leaders, from participating community gardens, and DUG staff will be contacted by phone or in-person to request their participation in the process evaluation interviews. Trained study staff will explain the outline of the interview and for those interested, schedule a time to meet at their respective garden or at a private room at the DUG offices. Informed consent forms will be available and will be reviewed by the University of Colorado Boulder Institutional Review Board. At the first interview, informed consent will be explained and signed. A copy will be made available at the participant's request.

XI. Process to Document Consent in Writing

See Section X.

XII. Procedures

Methods for data to be collected

<u>**Outcome Measures**</u>

The purpose of this study is to explore the relationship between participation in community gardens, diet, physical activity and weight status. Measures will be collected at baseline (T1: pre-gardening/March), at the height of harvest season (T2: Sept), and at the beginning of the next garden season (T3: pre-gardening/March). At each time point, study participants will complete three 24-hour dietary recalls over a 2 week period, a 6-day activity monitoring period using accelerometry, a health interview and an assessment of height, weight, and waist circumference collected by trained staff using a scale and stadiometer. Health interviews will assess processes that may mediate the relationships between garden participation and health behaviors. The office visit will take 45-60 minutes. Table 2 summarizes the data collection plan.

In response to the COVID-19 Pandemic, the following adjustments have been made to minimize risk for study participants traveling to data collection site and minimize risk for study personnel:

- All current participants will be offered health visits that occur via phone or video conference call to complete W3T3 data collection. These visits are in progress and will continue through the end of the W3T3 data collection. Specifically, participants will:
    - Receive a phone call from one of the data collectors and will be offered the opportunity to complete their final health visit via phone. They will be read a script explaining the changes and will only be scheduled for a virtual health visit after they provide us verbal consent for the changes outlined below.
    - Complete three 24-hour dietary recalls over a 2-week period over the phone (no change);
    - A health interview collected over the phone and using digital version of previously approved answer cards and documents (change from in-person to over-the-phone);
    - 6 day activity monitoring protocol will be modified to include a digital version (e.g., pdf) of the activity log in the event that we are not able to safely use an activity monitor (Activpal). A paper version will be made available upon request and will be mailed to participants requesting or who are unable to access the digital version;
    - Height and weight measurements will be self-reported at the time of the phone health visit and will be marked as self-reported (change from trained staff measurement to self-report and no waist circumference will be measured).

Diet Assessment

Estimates of 3-day average nutrient and food intake will be calculated from self-reports of foods actually consumed. Data will be collected using 3 telephone-administered 24-hour recall interviews, which, although not devoid of measurement error, are considered the "gold standard" for evaluating a nutrition intervention.(39) The University of South Carolina School of Public Health will conduct the 24-hour recalls. The Nutrient Data System for Research software licensed from the Nutrition Coordinating Center at the University of Minnesota will be employed to conduct the dietary interviews in English or Spanish. Interviewers will be a team of bilingual (English/Spanish) experienced registered dietitians specifically trained in using the multi-pass approach to conduct telephone interviews. The Nutrient Data System for Research is considered the state-of-the-art research software for conducting 24-hour recall. Portion estimation is facilitated by the use of a validated, 2-dimensional, food portion visual that will be mailed to participants,(40) and which we have used successfully in multiple studies in adults and adolescents.

Prior to data collection, study participants will undergo a brief training (10–15 min) on how to use the FPV to estimate portion sizes of commonly eaten foods. This will take place at T1 visit when accelerometers are mounted on the thigh and when height and weight are measured. The training incorporates life-sized plates, glasses and utensils and food models, in a hands-on experiential interchange.(41) Three 24-hour recalls will be collected at each measurement period, which generates a reliable estimation of macronutrient and food group intake.(39, 42-46) Interviews will be scheduled such that information will be collected on 2 weekdays and 1 weekend day to help balance known cyclical differences in intake patterns.(47) Interviews are assigned on randomly selected days, and cold calls are made to the study subject to minimize preparation that could bias recall.(39) The sampling window will be adequately large (3 weeks in length) to allow multiple attempts on multiple days to contact participants for interviews, which will maximize the likelihood of completing an interview. Each set of three 24-hour recalls will be averaged to form a 3-day average recall.

Social Desirability Scale. Diet assessment in this study relies on participants' self-reported dietary intake. Studies have shown that participants can be biased when they are reporting their dietary intake, and that these biases follow patterns related to participants' demographic factors, body size, and psychosocial factors. One factor found to be important is social desirability, a type of response bias that is the tendency of participants to answer questions in a way that will seem favorably by others (48). This can arise in dietary surveys when participants over or under report certain foods they perceive as healthy or unhealthy. We will use the Marlowe-Crowne 2(10) Social Desirability Scale to assess social desirability and will use the scale to determine participants' bias when analyzing their 24-hour dietary recalls (49). This scale will be given as an electronic REDCap instrument at T2 for all W2 and W3 participants.

Data processing. The Nutrient Data System for Research software provides information about nutrients, foods, and food groups at the level of foods, meals and days, which can be used to calculate Healthy Eating Index-2010 scores.(50) In this study we are interested in fruit (cups), vegetable (cups), soluble and insoluble fiber intake (g), total energy (kilocalories) and Healthy Eating Index-2010 scores although we will have the opportunity to examine other dietary outcomes. Fruit and vegetable intakes can include or exclude food such as French fries or juice, and diet measures will be energy-adjusted.(51),(52)

Physical Activity

Estimates of sedentary time and physical activity will be measured objectively using wrist-mounted accelerometers and subjectively using a self-report questionnaire included in the health survey and activity log during the week that accelerometers are worn. Accelerometers have become an important tool to objectively monitor activity in free-living conditions and can be worn on the wrist, hip or leg. Wrist-mounted accelerometers have similar accuracy compared to the hip,(53-55) and result in excellent compliance.(56, 57) Acceleration data can provide an objective measure of the types of activity (e.g. sit vs. walk), as well as the intensity, duration and frequency of activity throughout the day.(56),(58) We will use ActivPal (Scotland)),(59, 60) which has been validated for quantifying physical activity when worn on the thigh.(61) Self-report physical activity and sedentary time data will be collected using the validated Global Physical Activity Questionnaire (GPAQ) and previous day recall (PDR).(62, 63) The GPAQ collects participants' activity and time spent sitting over the previous 7 days across 4 domains, including occupation, transportation, home and recreation/leisure-time.

Height and Weight

We will collect anthropometric data to describe BMI and waist circumference among participants at T1, T2, and T3 at a private screening room at the Denver Urban Gardens office (northwest/central Denver). Height, weight, and waist circumference will be measured using standard protocols(64) by trained staff. Height will be measured to the closest 0.1 cm using a portable stadiometer (SECA 214; Seca Corp) while the participant is standing without shoes. Weight will be measured to the nearest 0.23 kg (0.5 lb.) on a digital platform scale (BWB-800AS Digital Scale; Tanita). Scales will be calibrated before each measurement. Waist circumference will be measured to the nearest 0.01 cm as described by the NCHS.(65)

In response to the COVID-19 Pandemic, weight measurements will be self-reported at the time of the phone health visit and will be marked as self-reported. This is a change from the original protocol of trained staff measurement. We will also remove waist circumference measurement requirement due to the inaccuracies of self-calculated waist circumference.

Data processing. Accelerometry data will be downloaded to a PC and analyzed using custom software (Matlab, v12.0, Mathworks, Natick, MA and R, v2.15.1 or ActiLife6 and SAS 9.4). Accelerometry data files will be analyzed for missing data, defined as a sequence of 60 minutes of zero acceleration. If missing data periods are found such that the total wear time/day is less than 600 minutes, the data for that day will not be used in the analysis. A minimum of 4 days of activity data will be required for analysis. If we find that data from a large number of participants (>10%) does not meet the requirements for inclusion, we will schedule additional data collection periods.

Qualitative interviews.

We will conduct semi-structured interviews at three time points: in March and April of Year 2, in September and October of Year 2, and in Year 4. The aims of these interviews are to: 1) elucidate the mechanisms underlying the differences found in diet and activity between gardeners and non-gardeners, and 2) to assess maintenance of outcomes for Waves 1 and 2. During year 2, 15 non-intervention and 15 intervention participants will be interviewed to explore the factors that impact gardeners' and non-gardeners' diet and activity patterns using a life course transition approach. Intervention participants will be selected to generate a diverse sample of gender, age, and extent of garden involvement . Non-gardening participants will be chosen to match gardeners in terms of their respective age and gender. The selected participants will be recruited for the qualitative interviews by phone. Participants will be asked to complete a second interview in the fall of year 2. During year 4, 30 additional intervention participants from Waves 1 and 2 will be randomly selected to assess maintenance of outcomes after the intervention and the quantitative measurements are completed. We will assess motivation and contextual issues for gardening, time spent gardening, and diet and activity patterns. Participants will receive an additional $20 gift card incentive for participation in the qualitative component of the study. We will audio record all of the semi-structured interviews to improve data capture and monitor

for quality control. A verbal consent clause has been added to the qualitative interview guides and will be obtained from all of the participants. The digital files will be transcribed by a paid transcription service and deleted once the analysis is completed. All recordings will be deleted on or before December 31st, 2023.

In addition, we will contact the participants by phone and ask them if they would be willing to participate in a follow-up interview. If they say yes, we will schedule a time for the interview. Trained interviewers who are members of the CAPS team will conduct the interviews. We will remind the participants that they completed a consent form at the beginning of their last interview, ask them to read the consent form again, and afterward they have read it, ask them if they have any questions about the research study or the consent process. Before we begin the interview, we will ask the participants if they agree for the interview to be recorded.

Process Evaluation

To understand the effectiveness of the intervention, we will collect data from the three groups involved in the intervention: participants, garden leaders, and DUG staff. We will collect these data at CP1, CP2, and CP3 in the garden, by phone, or at the DUG office. The process evaluation data will be measured through the following surveys: the garden involvement, control group, and garden leader surveys and DUG staff survey. These check points will occur in June, July/August, and November/December of each study year. These instruments have been developed by the study team and have been reviewed by grant partners, staff, and advisory team members. We will be audio recording 10% of the garden involvement and garden leader interviews for quality control analyses. A verbal consent clause has been added to the survey guides and will be obtained from the randomly selected group of participants (~10%). Tapes will be transcribed by the research team and erased once the transcriptions are checked for accuracy. All recordings will be erased on or before December 31st, 2020.

Study Participants in Garden Intervention Group.

Garden involvement surveys will aim to: 1) understand level of involvement and participation in the community gardens, 2) understand barriers that prevent people from participating in their community garden, and 3) identify issues or concerns participants may have about their participation in the community garden. The control group survey will aim to understand participant well-being throughout the summer and will be used to help with retention and maintain contact with the control group participants.

Garden Leaders.

Garden leader's surveys will aim to 1) understand the amount and quality of communication between garden leaders, gardeners, DUG staff, and the study team, 2) understand the level of involvement of garden leaders in recruiting garden participants, 3) identify how to increase community awareness of the CAPS study, 4) identify why garden leaders chose to participate in the study, and 4) offer garden leaders the opportunity to give feedback on their involvement with the study.

DUG Staff.

Denver Urban Gardens staff surveys will aim to 1) understand the level of involvement and how DUG staff support community gardeners, 2) identify potential barriers and DUG specific opportunities for community gardens, 3) understand general garden recruitment and retention rates, 4) offer DUG staff the opportunity to give feedback on their experience with their garden leaders, CAPS project, and study team.

Table 2: Summary of Data Collected on Study Participants

Measures	Time to Complete	T1	CP1	CP2	T2	CP3	T3	T4	T5
24-hour Diet Recalls	20 min	X			X		X		
7-day activity monitoring	7 day passive monitoring	X			X		X		
**ActivePal Activity Logs**	5-10 min/day for 7 days	X			X		X		
Weight and Height	5 min	X			X		X		
Demographic, race/ethnicity/SES	5 min	X			X		X		
**Health Survey**	45 min	X			X		X		
**Social Desirability Scale**	5 min				X				
**Garden Involvement Survey**	15-20 min		X	X					
**Control Group Survey**	5 min		X	X					
Qualitative interviews (selection of 60 participants)	1 hour							X	X
**Garden Leader Interview Survey**	20 min			X		X			
**Denver Urban Garden Staff Survey**	30 min			X		X			

Data Analysis Plan

Aim 1 Statistical Analysis: Using an intent to treat analysis and a general mixed model(66) to allow for missing data, we will fit the 3-day average recall measurements of dietary intake as the outcome. For fixed predictors, we will use time of measurement, an indicator variable for gardening, and the time by treatment interaction. We will evaluate the distribution of the jackknifed, studentized residuals of fruit and vegetable intake measures for normality. If the distribution is significantly skewed, an appropriate Box-Cox transformation will be applied. We will test for year by effect homogeneity, and combine responses across years if, as we expect, little difference occurs year by year. The model will include random effects terms for neighborhood, which will allow the model to account for differential, unmeasured differences for each neighborhood, which may include race, socio-economic status, language and culture. We will compare study participants with no missing data to those with missing data in an effort to see if differential response rates may have biased the study.

Because gardeners may see each other in the garden each day, their dietary intake may show a higher correlation than that of wait-listers. Thus, two possible nested variance models include the model with a neighborhood random effect only, and a model with an extra random term for garden effect. We will compare the fit of two models using a chi-squared log likelihood test, and by comparing the Akaike information criteria.

In the model with the most appropriate variance, we will conduct hypothesis testing using the Kenward-Roger test.(67) We will use sensitivity analysis to examine the potential confounding, mediating, or moderating activity of variables such as age, BMI, gender, language, race, and socio-economic status. We will provide group means, confidence intervals, and graphical descriptions of the data.

Both within-participant (time) and between-participant (intervention) effects are of interest. The planned sequence of hypothesis tests is as follows: 1) Test the time-by-treatment interaction at a Type I error rate of 0.04; 2) If there is no time-by-treatment interaction, then test the main effect of gardening at a Type I error rate equal 0.005. If the main effect of gardening is significant, we will stop testing; 3) If there is no main effect of treatment, then test the main effect of time using orthogonal polynomials to examine the linear and quadratic trends each at a Type I error rate of 0.0025. The hypothesis testing uses an alpha spending approach to assure a total experiment-wise error rate of 0.05.

In response to the COVID-19 Pandemic, we will use sensitivity analysis to qualitatively examine any differences between models with and without data collected during this time period.

Power and Sample Size. Estimates of means and variances for fruit and vegetable intake were based on our cross-sectional study in Denver.(6) We conducted a power analysis for the overall test of time by treatment. At a Type I error rate of 0.04, power for the Hotelling-Lawley test is estimated at 0.98 with 30 neighborhoods, and 109 people per randomization arm, for a total sample size of 218. Assuming loss to follow-up of 30%, the total sample size required will be 312 people, or 156 per randomization arm group. The power analysis accounts for correlation within gardens, and within neighborhoods.

Aim 2 Statistical Analysis: Sedentary time, minutes of MVPA, and waist circumference will be evaluated using a similar statistical analysis as described for dietary intake. BMI will be modeled as a function of intervention, HEI, sedentary time, MVPA, fruit and vegetable intake, total energy intake, and their interactions. This allows the trial to model different inputs collectively as they relate to BMI. No additional adjustment for multiple comparisons will be performed, so each aim will be tested at a Type I error rate of 0.05.

Power and Sample Size. Estimates for the power analysis were taken from a population-based Neighborhood Environments and Health Survey (NEHS) of 470 residents of Denver (Litt, PI).(4, 6) Community gardeners in the study (N=63) reported an average of 146.6 (std. dev. = 12.1) hours of sedentary time per week, while non-gardeners reported an average of 153 hours (std. dev. = 9.6). Community gardeners in the study (N=63) had an average BMI of 24, while non-gardeners had an average BMI of 27.

Power analysis was conducted both for sedentary time, and for BMI. With a Type I error rate of 0.04, the power for a Hotelling-Lawley test of the group by time interaction reflecting a decrease in *sedentary time* of 6.4 hours per week in midsummer for gardeners (std. dev = 12) will be more than 99% for 30 neighborhoods, and 120 people per randomization arm, for a total sample size of 240 people. Assuming loss to follow-up of 30%, the total sample size required to achieve that power will be 312 in 39 neighborhoods, or 156 per randomization arm. The power analysis accounts for correlation within gardens, and within neighborhoods. The power analysis proposed here is conservative, since it used the larger of two possible variance estimates. For the outcome of moderate-to-vigorous physical activity, we estimate adequate power, based on the difference in means from the NEHS (Litt, PI). Community gardeners reported an average of 2.5 more hours a week of moderate to vigorous physical activity, compared to non-gardeners.

Based on the preliminary *BMI* data, power for the hypothesis test of no difference in *BMI* slope between gardeners and non-gardeners would be over 0.999 for a sample size of 218 (312 before loss). The power analysis used Cohen's method to assess an increase in R^2 from 0.9614 to 0.9618.(68) The power analyses accounts for correlation within gardens, and within neighborhoods.

Aim 3 Statistical Analysis: The analyses for this aim will be carried out using Mplus(69) following well-established statistical methods for testing mediation,(70) particularly in a multilevel framework where individuals are nested within gardens/neighborhoods.(71) The potential mediators will be examined individually, as well as together in a multiple mediator model to account for relationships among mediators. The general structure of the proposed model is depicted in Figure 4 where the single mediator case is depicted for ease of presentation.

The regression of the potential mediators on community gardening will be estimated simultaneously with tests of the effect of the individual and neighborhood mediators on dietary intake and physical activity, controlling for gardening. The *a* path represents the impact of the community gardening intervention on the mediator and the *b* path represents the effect of the mediator on the health outcome. The *c'* path represents the effect of community gardening on the outcome after accounting for the mediator. The magnitude and significance of the individual paths and of the mediated effects will be assessed. The mediated effect will be calculated as the product of *a*b* and will be evaluated for statistical significance using asymmetric confidence limits.(72) Multilevel mediation analysis is feasible in Mplus;(71) depending on the magnitude of the garden-level ICCs observed in this study, the between and within components will either be modeled explicitly, or at least the standard errors will be corrected for the non-zero ICCs.(72) Effects are expected to be mediated by changes to the theoretical constructs from baseline to the first follow-up assessment. In the event that no effects of community gardening on health outcomes are observed in the analyses carried out for Aims 1 and 2, these analyses will instead focus on the effect of community gardening on the constructs and on relationships among constructs.

Figure 4: General Structure of Proposed Mediator Model

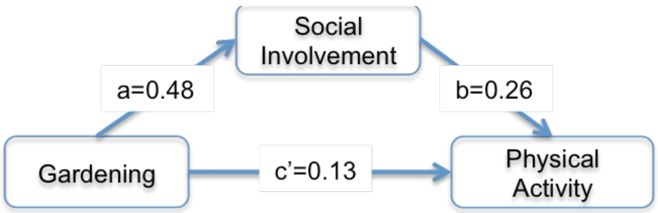

Power and sample size. Power calculations considered the magnitude of the individual paths, as well as power to test the mediated effect. Power was calculated in Mplus using Monte Carlo simulation.(73, 74) Estimates driving

the power analyses are shown in Figure 4 (presented as standardized beta coefficients) and are based on data from the GGHC study.(4, 6) This example estimate used social involvement as the mediator between community gardening and physical activity. Based on the prior study, power calculations were carried out assuming a neighborhood level ICC of 0.012 for physical activity and 0.0886 for fruit and vegetable intake. Assuming a two-sided $\alpha = 0.05$, the sample size of 240 participants will provide 80% power to detect the mediated effect for fruit and vegetable intake and 95% power to detect the mediated effect for physical activity. Power for the individual *a* and *b* paths was greater than 95% in all instances. We assume that the mediated effect in the proposed research will be at least as large in magnitude as those effects observed in the prior research, and may even be larger because the previous data were from a cross-sectional, observational study and measurement of physical activity and fruit and vegetable intake will be improved upon from the past work. These power estimates may also be considered conservative because the Monte Carlo simulations were not calculated using asymmetric confidence limits, which has been shown to be one of the most powerful methods for testing the significance of the mediated effect(75) and will be used in the proposed analyses.

Qualitative analysis. Analysis of the qualitative interviews will consist of thematic coding and comparative analyses of coded themes.(76) This analysis will inform the interpretation of mediators and maintenance (see Figure 3); deepen our understanding about the processes that shape diet and physical activity; and generate new insights about how socio-ecological interventions can prevent cancer among disadvantaged populations.

Figure 3: Proposed Timeline

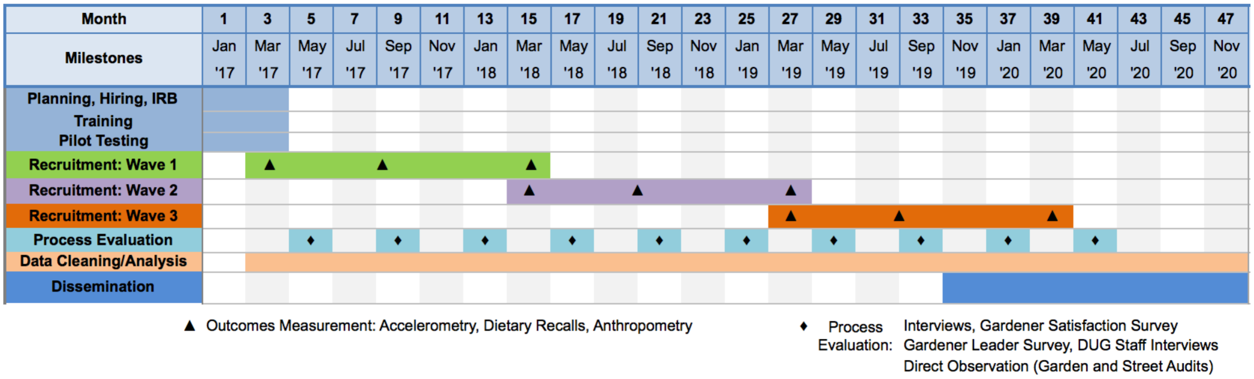

XII. Specimen Management
N/A

XIII. Data management
A REDCap database was created for participant recruitment that will contain names, phone numbers and email address and residential addresses. REDCap is a secure, HIPAA-compliant web-based application designed for data collection. The REDCap recruitment database will be maintained at the Colorado School of Public Health by trained staff. A unique study ID and randomization ID will be assigned to each participant.

Once randomization to study group has occurred, a new study ID number will be assigned, the PHI variables removed and exported into a separate study database. A file linking the original participant data to the study ID will be maintained for the duration of the project in a separate, password protected, encrypted file on the ColoradoSPH secure server. The study database will be maintained in in comprehensive password-protected database stored on a secured internal server at the CSPH. The ColoradoSPH uses network segregation as means of data separation as per HIPAA requirements. This network is disconnected from the standard, public university network through the use of firewalling and routers. Additionally, the ColoradoSPH uses a system of "access control" for certain folders that are located within the HIPAA network that will house the password-protected de-identified encounter level CHD data repository on a secure internal server. The folders are restricted and access is organized and granted by the IT department. Access to individual files and folders are assigned unique permissions stored in the Active Directory. The ColoradoSPH`s IT team will authorize members of the research team to have access to a specific folder housing the project data. Access to this folder will be restricted to the research team, and users will require passwords to access this folder. The password will be a nonsensical combination of numbers and letters, changed on a regular schedule, never repeated, and stored away from the computer.

No findings or study data will ever be linked to individual's identifying data and data will not be available to employers, individuals, or other outside parties except as mandated by law, or for research purposes upon completion of all IRB, ethics, and review procedures.

The confidentiality of eligible individuals who cannot be contacted for pre-screening, do not agree to participate or do not meet the study requirements will be maintained to determine the reach of the project. Information for these individuals will be stripped of direct and indirect identifiers, assigned a unique ID and stored in a HIPPA-compliant RedCAP database maintained at the Colorado School of Public Health by trained staff.

XIV. Withdrawal of Participants

There may be circumstances under which participants will be withdrawn from the study without consent. These include their inability to follow study procedures or comply with data collection procedures. In these instances, the investigator will ask the subject to continue participation in other research activities for this study such as obtaining data through direct observation and subject interviews as part of the process evaluation.

If and when subjects withdraw from this study, the investigator will document each instance of a subject's withdrawal and the reason for the withdrawal and the components of the trail that are affected by the withdrawal (all components, the diet component, or some combination of components).

XV. Risks to Participants

There are no psychological, social, or legal risks associated with participation in the study, other than the emotional stress that may be associated with discussions about health behaviors and weight status.

Potential Scientific Problems

The major concerns for this trial include: differential dropout and lack of compliance with data collection requirements. The study design should be robust to differential dropout for three reasons. First, a program is described above. Second, data from our survey of the targeted recruitment group shows that differential dropout is unlikely. In a 2011 survey of 50 Denver residents who were on a DUG waitlist, 70% of the 30 respondents said that they would be willing to stay on a waitlist for at least two years. 64% of respondents indicated that they would participate in a randomized controlled trial involving gardening, even if they could not garden during the study. This feasibility study will monitor compliance with our data collection protocol.

XVI. Management of Risks

See Section XVI and XIX.

XVII. Potential Benefits and Knowledge Gained:

Benefits include possibility for healthier lifestyles for residents (e.g., eating more fruits and vegetables, increased physical activity, reduction in sedentary behaviors, increased adherence to ACS Screening Guidelines), in accordance with Healthy People goals, the USDHHS National Prevention and Health Promotion Strategy and the American Cancer Society. Benefits will also include opportunity to receive health assessment information such as BMI. Participants will be compensated for their time during the visits with a small gift card incentive.

This research will shed light on the potential for impact of community interventions for the prevention of cancer and other chronic diseases. Thus, risks to study participants are reasonable in light of the knowledge that we expect to gain through this study.

Knowledge to be gained

The major importance of this study will be to understand how participation in a community garden-based intervention affects changes in health behaviors. Such an intervention will build on past cross-sectional research demonstrating the health and social benefits of community gardens and offer insights about the mechanisms that might explain these benefits.

XVIII Provisions to monitor data for the safety of participants

Process Evaluation: Reach, Dose, Fidelity, and Retention

The goals of the quality control protocol will be to 1) standardize the measurement techniques, 2) monitor the quality of measurements over the course of the study, and 3) to document the validity and precision of the measurements. The Principal and Co-Investigators will supervise all quality control efforts. Interviews, surveys and garden-specific direct observation in the garden will be used to assess the intervention. The evaluation components, informed by Linnan and others, are described below.[69-71]

1. Reach, Dose and Fidelity. Intervention delivery will be assessed through 1) **reach** (e.g. % eligible who enroll, who drops out and why, % who completed the T1-T6 assessments); 2) **dose** delivered and received (e.g. intensity of garden use, and uptake of garden programming including new gardener workshops, leadership training frequency); and 3) **fidelity** (% of people assigned to each arm who did not cross over to the other arm and whether the intervention was delivered in the way it was intended). We will also assess whether new gardener workshops were offered, whether seeds and plant starts were available, and whether social events were scheduled (fidelity). As described in Section XII. Procedures, gardener involvement surveys, collected by CAPS study staff, will assess participation in garden-specific workshops, tactics deployed by gardeners (advocacy and outreach), and activities for gardeners (gardener engagement). Garden leader surveys will assess DUG-specific garden leader roles and behaviors, and provide valuable garden management information, to be used to better enhance and promote the CAPS project and internal DUG programming and planning. The DUG staff survey will gather information about quality of interactions among CAPS staff, garden leaders, and DUG staff, which will be used to strengthen the research protocols and continue to integrate the information gathered through the study into current DUG practices. The information collected from these surveys are important for the sustainability of the program and for diffusion of the program knowledge to in practice in communities beyond Denver. Semi-structured interviews, which will be conducted on a subset of participants, will elicit more in-depth knowledge about participants' perceptions of the quality of the garden experience and their level of satisfaction. Direct observations will be conducted in the garden (at 6 and 12 weeks into gardening season) to monitor plot use and productivity, using previously developed and validated audits of garden plots and amenities.

2. Attrition. Study results could be adversely affected by poor adherence to the study protocol and could threaten the internal validity (e.g. differential attrition across intervention and control groups), although perfect compliance is not likely to be necessary to establish benefit. We recognize a few areas where poor adherence may be an issue. First, participants randomized to remain on waitlist may decide to garden elsewhere. Before randomization, staff will share expectations with participants about study conditions. We will ask participants whether they believe they can refrain from gardening elsewhere. Based on a wait list survey conducted in 2011, individuals on DUG waitlists were asked about their willingness to participate in a randomized controlled trial and the acceptability of being randomized to the garden plot or to waiting one season. Most participants (75%) indicated their support of the project and their willingness to participate. For this study, if individuals do not think they can keep this commitment, they will be excluded; if they respond positively, they will be enrolled.

3. Retention program. Once randomization occurs, study coordinators will maintain contact with both intervention and control participants through scheduled assessment periods (T1-T3) and interim phone calls (e.g., May, Sept, Nov, Jan) in order to maintain contact with study participants and minimize dropout and loss to follow-up. Incentives, which are described in Section IX, will be provided.

Once our application is reviewed, we will request an IRB Authorization Agreement (IAA) from Michigan State University, University of South Carolina, and UC Denver Anschutz. Eligibility screening worksheets and registration forms will be developed by CU-Boulder.

Trial Oversight

The principal investigator will be responsible for the conduct of this study, overseeing participant safety, executing the data and safety monitoring (DSM) plan, and complying with all reporting requirements to local and federal authorities. This oversight will be accomplished through additional oversight from the Data and Safety Monitoring Committee (DSMC) at the University of Colorado Cancer Center (CU Cancer Center). The DSMC is responsible for ensuring data quality and study participant safety for all trials at the CU Cancer Center. A summary of the DSMC's activities is as follows:

- Conduct of internal audits
- Has the authority to close and/or suspend studies for safety or conduct issues
- May submit recommendations for corrective actions to the CU Cancer Center's Executive Committee

Study audits conducted by the DSMC will consist of a review of the regulatory documents, consent forms, and source data verification. Documentation of the audit conducted by the DSMC will then need to be submitted to the IRB of record at the time of the IRB's continuing review of this trial.

XIX. Provisions to protect privacy interests of participants
N/A

XX. Medical care and compensation for injury
N/A

XXI. Cost to participants
We anticipate minimal costs to the participants. Compensation will be made to participants if costs accrue for cell phone usage to response to diet recall survey. We will monitor cell phone usage and charges through our process evaluation. Those assigned to gardens may incur costs to cover plant starts and seeds, if the donated seeds and transplants are not enough.

XXII. Drug administration
N/A

XXIII. Investigational devices
N/A

XXIV. Multi-Site Studies
Subject recruitment and data collection will be performed off site, in Denver, with 30 community gardens serving as the loci of the intervention and related monitoring and engagement. Data management will be conducted by study personnel and will have input by key personnel involved in the research at the University of Colorado Boulder (Litt), University of Colorado Anschutz (Crume, Glueck, Hamman), Michigan State University (Alaimo), Colorado State University (Li), and the University of South Carolina (Hebert) (See Section XIII for more information).

XXV. Sharing of results with participants
Research results will be shared with study participants at the end of the study.

XXVI. Literature Cited

1. McCormack L, Laska M, Larson N, Story M. Review of the nutritional implications of farmers' markets and community gardens: A call for evaluation and research efforts. Journal of the American Dietetic Association. 2010;110:399-408.
2. Colorado Department of Public Health and Environment. Cancer and Poverty: Colorado 2001-2012. Denver, CO; 2013.
3. Krieger N, Williams DR, Moss NE. Measuring Social Class in US Public Health Research: Concepts, Methodologies, and Guidelines. Annual Review of Public Health. 1997;18:341-78.
4. Comstock N, Dickinson M, Marshall JA, Soobader MJ, Turbin MS, Buchenau M, et al. Neighborhood Attachment And Its Correlates: Exploring Neighborhood Conditions, Collective Efficacy And Gardening. Journal of Environmental Psychology. 2010;30:435-42.
5. Hale J, Knapp C, Bardwell L, Buchenau M, Marshall J, Sancar F, et al. Connecting food environments and health through the relational nature of aesthetics: Gaining insight through the community gardening experience. Social Science & Medicine. 2011;72:1853-63.
6. Litt JS, Soobader M, Turbin MS, Hale J, Buchenau M, Marshall JA. The influences of social involvement, neighborhood aesthetics and community garden participation on fruit and vegetable consumption. The American Journal of Public Health. 2011;101:1466-73.
7. Teig E, Amulya J, Buchenau M, Bardwell L, Marshall J, Litt JS. Collective efficacy in Denver, Colorado: Strengthening neighborhoods and health through community gardens. Health and Place. 2009;15:1115-22.
8. Alaimo K, Packnett E, Miles RA, Kruger DJ. Fruit and vegetable intake among urban community gardeners. Journal of Nutrition Education and Behavior. 2008;40(2):94-101.

9. Alaimo K, Reischl TM, Ober-Allen J. Community gardening, neighborhood meetings and social capital. Journal of Community Psychology. 2010;38(4):497-514.
10. Allen JO, Alaimo K, Elam D, Perry E. Growing Vegetables and Values: Benefits of Neighborhood-Based Community Gardens for Youth Development and Nutrition. Journal of Hunger and Environmental Nutrition. 2008;3(4):418-39.
11. Bronfenbrenner U. The ecology of human development: Experiments by nature and design. Cambridge, Mass.: Harvard University Press; 1979. xv, 330 p. p.
12. Bandura A. Social Foundations of Thought and Action: A Social Cognitive Theory. Englewood Cliffs, NJ: Prentice Hall; 1986.
13. Kushi L, Doyle, C., McCullough, M., et al.,. American Cancer Society Guidelines on Nutrition and Physical Activity for Cancer Prevention. CA: A Cancer Journal for Clinicians. 2012;62(1):30-67.
14. American Cancer Society. Cancer Facts & Figures 2014. Atlanta, GA; 2014.
15. National Health Statistics Group. National Health Expenditure Fact Sheet. Baltimore, MD: Centers for Medicare and Medicaid Services; 2009.
16. Centers for Disease Control and Prevention. Chronic Diseases: The Power to Prevent, The Call to Control: At A Glance 2009 Atlanta: CDC; 2011 [Available from: http://www.cdc.gov/chronicdisease/resources/publications/aag/chronic.htm.
17. Byers TE, Wolf HJ, Bauer KR, Bolick-Aldrich S, Chen VW, Finch JL, et al. The impact of socioeconomic status on survival after cancer in the United States. Cancer. 2008;113(3):582-91.
18. Roger VL, Go AS, Lloyd-Jones DM, Adams RJ, Berry JD, Brown TM, et al. Heart Disease and Stroke Statistics: 2011 Update Circulation. 2011;123(4):e18-e209.
19. Centers for Disease Control and Prevention. Annual smoking-attributable mortality, years of potential life lost, and economic costs --- United States, 1995--1999. Morbidity and Mortality Weekly Report. 2002;51(14):300-3.
20. Ness AR, Powles JW. Fruit and vegetables, and cardiovascular disease: a review. International Journal of Epidemiology. 1997;26(1):1-13.
21. Pate RR, Pratt M, Blair SN, Haskell WL, Macera CA, Bouchard C, et al. Physical activity and public health. A recommendation from the Centers for Disease Control and Prevention and the American College of Sports Medicine. JAMA: The Journal of the American Medical Association. 1995;273(5):402-7.
22. Smedley BD, Stith AY, Nelson AR. Unequal treatment: Confronting racial and ethnic disparities in health care. Washington, DC: Institute of Medicine 2003.
23. Mensah GA, Mokdad AH, Ford ES, Greenlund KJ, Croft JB. State of Disparities in Cardiovascular Health in the United States. Circulation. 2005;111(10):1233-41.
24. Mensah GA. Eliminating Disparities in Cardiovascular Health. Circulation. 2005;111(10):1332-6.
25. Smedley BD, Syme SL. A social environmental approach to health and health interventions. Promoting Health: Intervention Strategies from Social and Behavioral Research. 1st ed. Washington, DC: National Academy Press; 2000.
26. Hayman L, Hughes S. Cardiovascular Health for All. Journal of Cardiovascular Nursing. 2006;21(2):154-5.
27. Institute of Medicine. How far have we come in reducing health disparities?: Progress since 2000: Workshop summary. Washington, DC: The National Academies Press; 2012.
28. U. S. Census Bureau. American Community Survey. 2008.
29. Blair D, Giesecke CC, Sherman S. A Dietary, Social and Economic Evaluation of the Philadelphia Urban Gardening Project. The Journal of Nutrition Education. 1991;23(161):167.
30. Armstrong D. A Survey of Community Gardens in Upstate New York: Implications for Health Promotion and Community Development. Health & Place. 2000;6:319-27.
31. Kinney AY, Bloor LE, Martin C, Sandler RS. Social Ties and Colorectal Cancer Screening among Blacks and Whites in North Carolina. Cancer Epidemiology Biomarkers & Prevention. 2005;14(1):182-9.
32. Litt JS, Schmiege SJ, Hale JW, Buchenau M, Sancar F. Exploring ecological, emotional and social levers of self-rated health for urban gardeners and non-gardeners: A path analysis. Social Science & Medicine. 2015;144(November):1-8.
33. Hoyert D, Kochanek K, Murphy S. Deaths: Final data for 1997. National Vital Statistics Report. National Center for Health Statistics; 1999 June 30. Report No.: Vol. 47, No. 19.
34. Blacksher E, Lovasi G. Place-focused physical activity research, human agency, and social justice in public health: Taking agency seriously in studies of the built environment. Health and Place. 2012;18(2):172-9.
35. Wallerstein N, Yen, IH, Syme, SL,. Integration of Social Epidemiology and Community-Engaged Interventions to Improve Health Equity. American Journal of Public Health. 2011;101(5):822-30.

36. McCullough ML, Patel AV, Kushi LH, Patel R, Willett WC, Doyle C, et al. Following cancer prevention guidelines reduces risk of cancer, cardiovascular disease, and all-cause mortality. Cancer Epidemiology, Biomarkers and Prevention. 2011;20:1089-97.
37. Ammerman AS, Lindquist CH, Lohr KN, Hersey J. The Efficacy of Behavioral Interventions to Modify Dietary Fat and Fruit and Vegetable Intake: A Review of the Evidence. Preventive Medicine. 2002;35(1):25-41.
38. Thomson C, Ravia J. A Systematic Review of Behavioral Interventions to Promote Intake of Fruit and Vegetables. Journal of the American Dietetic Association. 2011;111(10):1523-35.
39. Hebert JR, et al. Systematic Errors in Middle-Aged Women's Estimates of Energy Intake. Comparing Three Self-Report Measures to Total Energy Expenditure from Doubly Labeled Water. Annals of Epidemiology 2002;12(8):577-86.
40. Posner BM, Smigelski C, Duggal A, Morgan JL, Cobb J, Cupples LA. Validation of two-dimensional models for estimation of portion size in nutrition research. Journal of the American Dietetic Association. 1992;92(6):738-41.
41. Wilcox S, Sharpe P, Parra-Medina D, Granner M, Hutto B. A randomized trial of a diet and exercise intervention for overweight and obese women from economically disadvantaged neighborhoods: Sisters Taking Action for Real Success (STARS). Contemporary Clinical Trials. 2011;32(6):931-45.
42. Buzzard IM, et al. Monitoring dietary change in a low-fat diet intervention study: advantages of using 24-hour dietary recalls vs food records. Journal of the American Dietetic Association. 1996;96(6):574-9.
43. Hebert JR, et al. A comparison of selected nutrient intakes derived from three diet assessment methods used in a low-fat maintenance trial. Public Health Nutrition. 1998;1(3):207-14.
44. Nutrition Consulting Enterprises. The 2D Food Portion Visual. Framingham, MA1996.
45. Resnicow K, Odom E, Wang T, Dudley WN, Mitchell D, Vaughan R, et al. Validation of three food frequency questionnaires and 24-hour recalls with serum carotenoid levels in a sample of African-American adults. American Journal of Epidemiology. 2000;152(11):1072-80.
46. Yaroch A, Tooze J, Thompson F. Evaluation of Three Short Dietary Instruments to Assess Fruit and Vegetable Intake: The National Cancer Institute's Food Attitudes and Behaviors Survey. Journal of the Academy of Nutrition and Dietetics. 2012;112(10):1570-7.
47. Basiotis PP, et al. Number of days of food intake records required to estimate individual and group nutrient intakes with defined confidence. Journal of Nutrition. 1987;117(9):1638-41.
48. Hebert JR, Hurley TG, Peterson KE, Resnicow K, Thompson FE, Yaroch AL, et al. Social desirability trait influences on self-reported dietary measures among diverse participants in a multicenter multiple risk factor trial. The Journal of Nutrition. 2008;138(1):226S-34S.
49. Strahan R, Gerbasi KC. Short, homogeneous versions of the Marlowe-Crowne social desirability scale. Journal of Clinical Psychology. 1972.
50. Guenther P, Casavale K, Reedy J, Kirkpatrick S, Hiza H, Kuczynski K, et al. Update of the Healthy Eating Index: HEI-2010. Journal of the Academy of Nutrition and Dietetics. 2013;113(4):569-80.
51. Sievert Y, Schakel S, Buzzard I. Maintenance of a nutrient database for clinical trials. Control Clinical Trials. 1989;10(4):416-25.
52. Hébert JR, Hurley TG, Steck SE, Miller DR, Tabung FK, Peterson KE, et al. Considering the Value of Dietary Assessment Data in Informing Nutrition-Related Health Policy. Advances in Nutrition: An International Review Journal. 2014;5(4):447-55.
53. Matthews CE, Hagstromer M, Pober DM, Bowles HR. Best practices for using physical activity monitors in population-based research. Med Sci Sports Exerc 2012;44(1S):S68-76.
54. Esliger DW, Rowlands AV, Hurst TL, Catt M, Murray P, Eston RG. Validation of the GENEA Accelerometer. Medicine & Science in Sports & Exercise. 2011;43(6):1085-93.
55. Zhang S, Rowlands AV, Murray P, Hurst TL. Physical Activity Classification Using the GENEA Wrist-Worn Accelerometer. Medicine & Science in Sports & Exercise. 2012;44(4):742-8.
56. Ward V, Schaefer C, Lampe S, Nace H, E. K, Brink L, et al. Achieving Excellent Compliance in Multi-day Free-living Activity Monitoring in Elementary School Students: The IPLAY Study. Medicine & Science in Sports & Exercise. 2011;42(5).
57. Troiano RP, Berrigan D, Dodd KW, Mâsse LC, Tilert T, McDowell M. Physical Activity in the United States Measured by Accelerometer. Medicine & Science in Sports & Exercise. 2008;40(1):181-8.
58. Butte NF, Ekelund U, Westerterp KR. Assessing physical activity using wearable monitors: measures of physical activity. Medicine & Science in Sports & Exercise. 2012;44(1S):S5-S12.
59. Ozemek C, Kirschner, M. M., Wilkerson, B. S., Byun, W., & Kaminsky, L. A., . Intermonitor reliability of the GT3X+ accelerometer at hip, wrist and ankle sites during activities of daily living. Physiological Measurement,. 2014;35:129-38.

60. Trost SG, Zheng, Y., & Wong, W. K.,. Machine learning for activity recognition: Hip versus wrist data. . Physiological Measurement,. 2014;35:2183-9.
61. Esliger DW, Rowlands AV, Hurst TL, Catt M, Murray P, Eston RG. Validation of the GENEA Accelerometer. Medicine & Science in Sports & Exercise. 2010.
62. Craig C, Marshall A, Sjostrom M. International physical activity questionnaire: 12-country reliability and validity. Medicine & Science In Sports & Exercise. 2003;35:1381-95.
63. Matthews C, Keadle, SK, Sampson, J, Lyden, K, Bowles, H, Moore, S, Libertine, A, Freedson, P, Fowke, J,. Validation of a Previous-Day Recall Measure of Active and Sedentary Behaviors. Med Sci Sport Exerc. 2013;45(8):1629-38.
64. Malina RM. Anthropometry. In: Maud P.J., Foster C., editors. Physiological Assessment of Human Fitness. Champaign, IL: Human Kinetics; 1995. p. 205-19.
65. National Center for Health Statistics. Anthropometric Procedures Videos - Circumferences Atlanta, GA2011 [Available from: http://www.cdc.gov/nchs/video/nhanes3_anthropometry/circumference/circumference.htm.
66. Laird NM, Ware J. Random-effects models for longitudinal data. Biometrics. 1982;38(4):963–74.
67. Kenward M, Roger J. Small sample inference for fixed effects from restricted maximum likelihood. Biometrics. 1997;53(3):983-97.
68. Cohen J. Statistical power analysis for the behavioral sciences. 2nd ed. Hillsdale, NJ: Erlbaum; 1988.
69. Muthén LK, Muthén BO. Mplus User's Guide: Sixth Edition. In: Muthén M, editor. Los Angeles, CA1998.
70. MacKinnon DP, Lockwood CM, Hoffman JM, West SG, Sheets V. A comparison of methods to test mediation and other intervening variable effects. Psychological Methods. 2002;7(1):83-104.
71. Preacher K, Zyphur M, Zhang Z. A general multilevel SEM framework for assessing multilevel mediation Psychological Methods. 2010;15:209-33.
72. MacKinnon DP, Fritz, M. S., Williams, J., & Lockwood, C. M. Distribution of the product confidence limits for the indirect effect: Program PRODCLIN. Behavior Research Methods. 2007;39:384-9.
73. Thoemmes F, MacKinnon DP, Reiser MR. Power Analysis for Complex Mediational Designs Using Monte Carlo Methods. Structural Equation Modeling: A Multidisciplinary Journal. 2010;17(3):510-34.
74. Muthén LK, Muthén BO. How to use a Monte Carlo study to decide on sample size and determine power. Structural Equation Modeling. 2002;4:599-620.
75. Fritz MS, MacKinnon DP. Required sample size to detect the mediated effect. Psychological Science. 2007;18:223-39.
76. Miles MB, Huberman AM. Qualitative Data Analysis: An Expanded Sourcebook. Thousand Oaks, California: Sage Publications; 1994.
